# Relaxation of the Plant Cell Wall Barrier via Zwitterionic Liquid Pretreatment for Micelle‐Complex‐Mediated DNA Delivery to Specific Plant Organelles

**DOI:** 10.1002/anie.202204234

**Published:** 2022-06-24

**Authors:** Takaaki Miyamoto, Kousuke Tsuchiya, Kiminori Toyooka, Yumi Goto, Ayaka Tateishi, Keiji Numata

**Affiliations:** ^1^ Biomacromolecules Research Team RIKEN Center for Sustainable Resource Science Saitama 351-0198 Japan; ^2^ Department of Material Chemistry Graduate School of Engineering Kyoto University Kyoto-Daigaku-Katsura, Nishikyo-ku Kyoto 615-8510 Japan; ^3^ Technology Platform Division Mass Spectrometry and Microscopy Unit RIKEN Center for Sustainable Resource Science Yokohama 230-0045 Japan

**Keywords:** Gene Technology, Micelles, Nanotechnology, Peptides, Zwitterions

## Abstract

Targeted delivery of genes to specific plant organelles is a key challenge for fundamental plant science, plant bioengineering, and agronomic applications. Nanoscale carriers have attracted interest as a promising tool for organelle‐targeted DNA delivery in plants. However, nanocarrier‐mediated DNA delivery in plants is severely hampered by the barrier of the plant cell wall, resulting in insufficient delivery efficiency. Herein, we propose a unique strategy that synergistically combines a cell wall‐loosening zwitterionic liquid (ZIL) with a peptide‐displaying micelle complex for organelle‐specific DNA delivery in plants. We demonstrated that ZIL pretreatment can enhance cell wall permeability without cytotoxicity, allowing micelle complexes to translocate across the cell wall and carry DNA cargo into specific plant organelles, such as nuclei and chloroplasts, with significantly augmented efficiency. Our work offers a novel concept to overcome the plant cell wall barrier for nanocarrier‐mediated cargo delivery to specific organelles in living plants.

## Graphical abstract

A unique strategy to overcome the plant cell wall barrier for organelle‐specific DNA delivery is presented. This strategy leverages the synergistic effects of a cell wall‐loosening zwitterionic liquid and an organelle‐targeting micelle complex, allowing greatly augmented DNA delivery to target organelles, such as chloroplasts, in living plants.

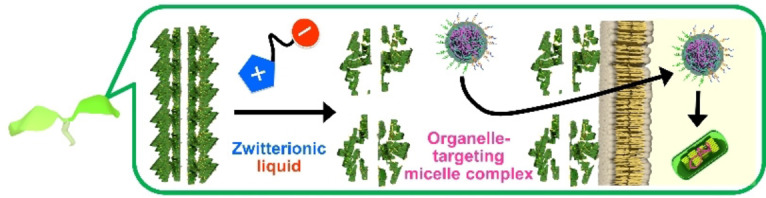

## Introduction

Nanoscale carriers enabling intracellular cargo delivery have emerged as a promising tool to modulate and probe plant function.^[1]^ Plant genetic engineering achieved by the delivery of genetic cargoes (e.g., DNA) provides opportunities to improve crop yield, enhance pharmaceutical biosynthesis, and study gene function.^[2]^ Nanocarrier‐mediated DNA delivery offers several advantages over conventional delivery methods using host species‐limited Agrobacterium infection and specialized equipment‐required particle bombardment.^[3]^ For example, nanocarriers are capable of delivering DNA cargo without specialized equipment in a plant species‐independent manner.^[4]^ Notably, some nanocarriers, including peptide‐based nanocomplexes and carbon nanotubes (CNTs), have enabled plasmid DNA (pDNA) delivery to specific organelles (i.e., chloroplasts and mitochondria) in intact plants,^[5]^ whereas conventional methods cannot selectively target these organelles. Since plant photosynthesis and respiration take place in chloroplasts and mitochondria, respectively, genetic modification of these organelles through pDNA delivery is essential for fundamental plant biology and various agronomic applications. However, successful organelle‐specific pDNA delivery remains limited, and the delivery efficiency must be improved to facilitate organelle transformation.

The primary plant cell wall poses a formidable and dynamic barrier to nanocarrier‐mediated cargo delivery into plant cells. The cell wall permeation ability of nanocarriers varies with plant species, tissue type, and nanocarrier properties, including size, shape, charge, and hydrophobicity.^[6]^ However, previous studies have suggested that spherical nanoparticles larger than 50 nm tend to exhibit limited cell wall permeation ability.^[7]^ Nanocarriers need to form a stable complex with the pDNA cargo to guide it into specific subcellular compartments without degradation. Such a complex is generally larger than 50 nm, even when the pDNA is condensed through electrostatic interactions,^[4g]^ and thus may have difficulty permeating the cell wall efficiently. A conventional approach to avoiding the cell wall barrier is the polyethylene glycol‐mediated transformation of protoplasts,^[8]^ plant cells whose cell wall is removed by enzymatic degradation. However, this approach suffers from the difficulty of regenerating entire plants from protoplasts, limiting its widespread use in plant transformation. An alternative approach to relaxing the size exclusion limit of the cell wall is necessary for boosting nanocarrier‐mediated pDNA delivery to target organelles.

We envision enhancing the permeability of the plant cell wall by disrupting the hydrogen bonds between the cell wall components. In the cell wall, hydrogen bonds direct the assembly of cellulose microfibrils from multiple cellulose chains. Hydrogen bonds also mediate the physical crosslinking between cellulose microfibrils and hemicellulose polymers to form a complex network.^[9]^

Ionic liquids (ILs), defined as organic salts with melting points <100 °C, have been utilized for cell wall deconstruction due to their ability to interact with cellulose via hydrogen bonding.^[10]^ However, ILs generally show cytotoxicity through perturbation of the plasma membrane.^[11]^ Recently, Kuroda et al. reported a low‐toxicity IL with a zwitterionic structure, in which an imidazolium cation is covalently tethered to a carboxylate anion.^[12]^ This zwitterionic IL (ZIL) can dissolve cellulose by disrupting intermolecular hydrogen bonds while being compatible with the plasma membrane. Despite these attractive properties, studies to date have not examined the impact of ZIL on cell wall permeability in living plants. Additionally, ZIL has never been explored for its use in nanocarrier‐mediated cargo delivery to plant organelles.

**Figure 1:**
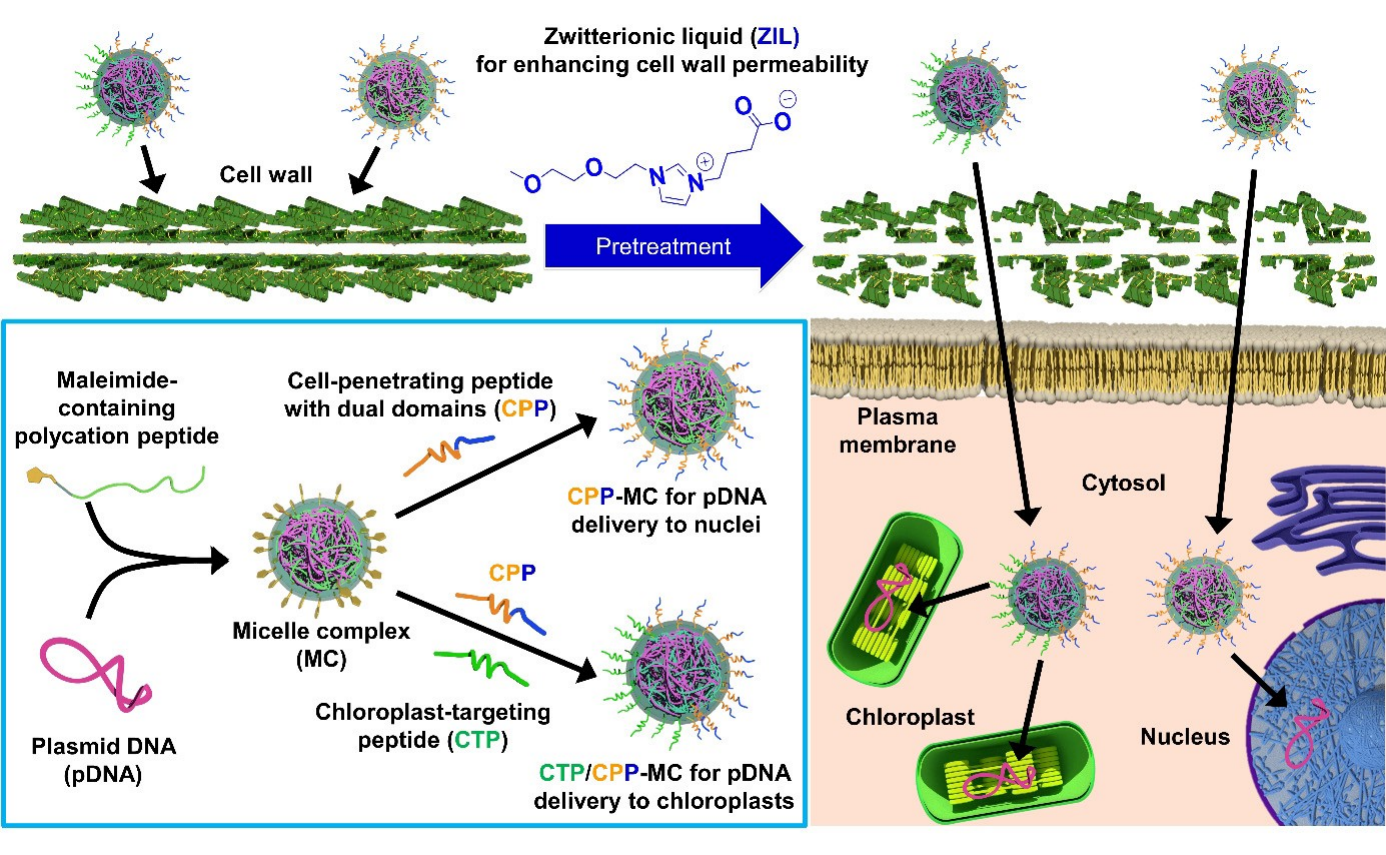
Schematic overview of pDNA delivery to specific organelles in plants through the combination of a zwitterionic liquid and peptide‐displaying micelle complexes. Zwitterionic liquid pretreatment is intended to enhance cell wall permeability, allowing the efficient translocation of peptide‐displaying micelle complexes across the cell wall into intracellular target organelles.

Herein, we describe an approach that leverages the synergistic effect of a cell wall‐loosening ZIL and an organelle‐targeting nanocarrier for pDNA delivery to specific plant organelles (Figure 1). In this approach, ZIL pretreatment is intended to increase the cell wall permeability by disrupting hydrogen bonds between the cell wall components. This can allow the nanocarrier to translocate efficiently across the cell wall and transfect the pDNA cargo into target organelles of living plants. We employed a peptide‐displaying micelle complex as the nanocarrier. In this micelle complex, cationic peptides condense the pDNA cargo to form the core, and peptides with cell‐penetrating and chloroplast‐targeting abilities are present at the surface. Cell‐penetrating peptides (CPPs) have been used for intracellular cargo transport through endocytic pathways across the plasma membranes of various types of plants,^[13]^ while chloroplast‐targeting peptides (CTPs) have enabled chloroplast‐specific cargo delivery by exploiting plant biorecognition systems.[^5c^, ^14^] Accordingly, CPP‐ and CTP/CPP‐displaying micelle complexes (CPP‐MC and CTP/CPP‐MC) can guide the pDNA cargo to plant nuclei and chloroplasts, respectively. We show for the first time that ZIL pretreatment can dissolve crystalline cellulose and promote cell wall permeability without causing cell death in a model plant system. Due to the benefits of ZIL pretreatment, CPP‐MC and CTP/CPP‐MC significantly improved pDNA delivery to the nucleus and chloroplast, respectively. This study provides a unique concept that combines ZIL and nanocarriers to overcome the otherwise impregnable barrier of the plant cell wall for organelle‐specific cargo delivery in living plants.

## Results and Discussion

### Biocompatibility of ZIL with Plants

**Figure 2:**
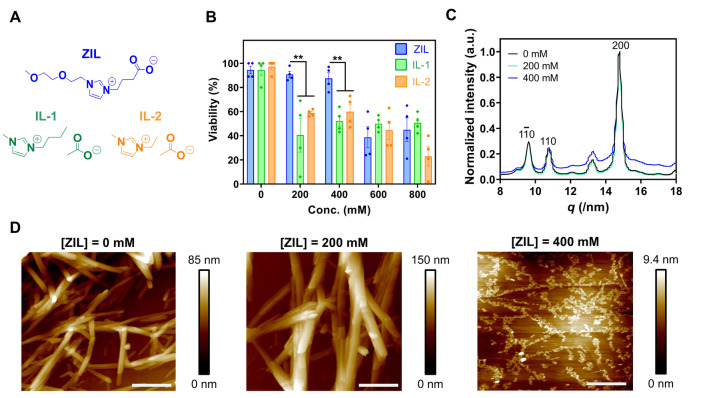
Biocompatibility and cellulose‐dissolving ability of ZIL. A) Chemical structures of ZIL, IL‐1, and IL‐2. B) Viability of *A. thaliana* seedlings pretreated for 3 h with various concentrations of ZIL, IL‐1, or IL‐2, followed by 24 h of incubation. The viability was determined by the Evans blue assay. Data from four biological replicates are represented as the mean±standard error values. Statistical significance: * *P*<0.05, ** *P*<0.01 based on Dunnett's T3 test (*n*=4). C) 1D WAXD profiles obtained from cellulose microcrystals 2 h after pretreatment with 0, 200, or 400 mM ZIL. D) AFM height images of cellulose microcrystals pretreated with 0, 200, or 400 mM ZIL for 2 h on a highly oriented pyrolytic graphite (HOPG) substrate. Scale bars, 500 nm. Color bars represent the height of the cellulose microcrystal.

We evaluated the cytotoxicity of ZIL and commercially available ILs (IL‐1 and IL‐2, Figure 2A) to seedlings of *Arabidopsis thaliana*, a model dicot plant system. The whole seedling was soaked in an aqueous solution containing ZIL, IL‐1, or IL‐2 at various concentrations for pretreatment. After pretreatment for 3 h followed by 24 h of incubation, the viability of seedlings was determined by Evans blue assay. ZIL at 200 and 400 mM exhibited negligible toxicity against seedlings, although IL‐1 and IL‐2 above 200 mM caused significant cell death at 24 h after pretreatment (Figure 2B). Seedlings pretreated with 200 and 400 mM ZIL grew as well as untreated control samples over 20 days whereas pretreatment with 200 and 400 mM ILs resulted in plant death (Figure S1). These results indicate that ZIL below 400 mM has no negative effects on plant growth, unlike commercially available ILs. The toxicity of ILs is most likely exerted by the disruption of plasma membranes.^[11]^

In contrast, the high compatibility of ZIL with plasma membranes has been suggested by both experimental data on bacteria and molecular dynamics simulations using a model lipid bilayer.^[15]^ The structure of ZIL, in which cationic and anionic moieties are covalently tethered, can suppress its interactions with plasma membranes, resulting in its lower toxicity against living plants compared with ILs.

### Dissolution of Microcrystalline Cellulose via ZIL Pretreatment

We examined the effect of ZIL on the crystalline structure of cellulose microcrystals from *Halocynthia sp*., which mainly adopt a cellulose Iβ form found in plant cell walls,^[16]^ by wide‐angle X‐ray diffraction (WAXD) measurements. The untreated cellulose microcrystals exhibited 200, 110, and 1‐10 reflection peaks (Figure 2C), a characteristic WAXD pattern of cellulose I structures.^[17]^ An almost identical WAXD pattern to that of untreated microcrystals was observed in those pretreated with 200 mM ZIL for 2 h (Figure 2C), suggesting that ZIL pretreatment at 200 mM did not affect the cellulose crystal structure. In contrast, an amorphous halo appeared in the cellulose microcrystals pretreated with 400 mM ZIL for 2 h (Figure 2C). The degree of crystallinity of cellulose microcrystals decreased from 89 % to 55 % after ZIL pretreatment at 400 mM. The morphology of the cellulose microcrystals was observed by atomic force microscopy (AFM) on a highly oriented pyrolytic graphite (HOPG) substrate after 2 h of pretreatment with 0, 200, or 400 mM ZIL. Pretreatment with 400 mM ZIL led to a significant disruption of cellulose microcrystal bundles, whereas pretreatment with 0 and 200 mM ZIL caused no morphological changes (Figure 2D and S2). In summary, ZIL can partially dissolve cellulose microcrystals at 400 mM, and this concentration caused no cytotoxicity to plants (Figure 2B).

### Enhanced Cell Wall Permeability via ZIL Pretreatment

**Figure 3:**
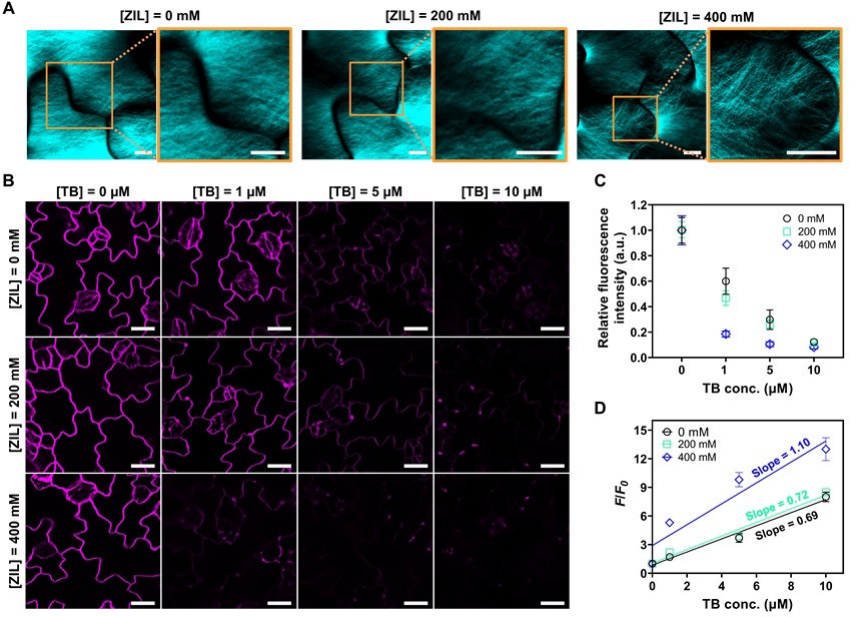
Effect of ZIL on cell wall permeability in plants. A) CLSM images of cellulose fibrils stained with calcofluor white in epidermal cells of *A. thaliana* cotyledons after pretreatment with 0, 200, or 400 mM ZIL for 3 h. Scale bars, 5 μm. B) CLSM images showing FM4‐64 fluorescence quenching by TB in epidermal cells of *A. thaliana* cotyledons pretreated for 3 h with 0, 200, or 400 mM ZIL. Scale bars, 20 μm. C) Relative fluorescence intensity of FM4‐64 at several TB concentrations shown in B. Data from four biological replicates are represented as the mean±standard error values. D) Stern–Volmer plots of FM4‐64 fluorescence quenching by TB in epidermal cells of *A. thaliana* cotyledons pretreated for 3 h with 0, 200, or 400 mM ZIL. The slope of the regression line indicates the quenching efficiency. Data from four biological replicates are represented as the mean ± standard error values.

To investigate the effect of ZIL pretreatment on the cell walls of living plants, we performed confocal laser scanning microscopy (CLSM) on *A. thaliana* cotyledons pretreated for 3 h with 0, 200, or 400 mM ZIL followed by staining with calcofluor white, a fluorescent dye that binds to cellulose in the cell walls. Cellulose microfibrils were similarly visualized by calcofluor white in the CLSM images of cotyledons pretreated with 0 and 200 mM ZIL (Figure 3A). Conversely, pretreatment with 400 mM ZIL appeared to decrease the density of cellulose microfibrils in some regions of the cell walls, although these decreases were not uniformly distributed throughout the cotyledon cells (Figure 3A). We also observed the cell walls of ZIL‐pretreated *A. thaliana* cotyledons using transmission electron microscopy (TEM) and field emission‐scanning electron microscopy (FE‐SEM). According to the TEM images, the morphology of the cell wall was maintained after ZIL pretreatment (Figure S3A). However, the cell wall density in some areas was likely decreased by pretreatment with 400 mM ZIL (Figure S3A). The FE‐SEM images indicated the loosening of leaf cuticle in some regions after pretreatment with 400 mM ZIL (Figure S3B). These observations suggest partial disruption of the cell wall and leaf cuticle by 400 mM ZIL pretreatment.

To further evaluate the effect of ZIL on cell wall permeability, we performed a fluorescence quenching assay using a combination of FM4‐64, a fluorescent dye staining the plasma membranes, and trypan blue (TB), a quencher for FM4‐64, in reference to a study by Liu et al.^[18]^ This quenching assay allows quantification of the cell wall permeability in living plants by estimating the quenching efficiency of extracellular TB toward the plasma membrane‐binding FM4‐64: the high cell wall permeability is represented by the high quenching efficiency and vice versa.

For the quenching assay, the cotyledons were pretreated for 3 h with ZIL (0, 200, or 400 mM), stained for 3 min with FM4‐64 (50 μM), and incubated for 3 min with several concentrations (0–10 μM) of TB prior to CLSM observations. The intensity of FM4‐64 fluorescence on the plasma membranes decreased with increasing concentrations of TB in cotyledons pretreated with ZIL (Figure 3B and C). The quenching efficiency of each system was estimated from the Stern–Volmer plots of FM4‐64 fluorescence quenching with TB (Figure 3D), where the slope of the regression line corresponds to the quenching efficiency. The cotyledons pretreated with 400 mM ZIL exhibited higher quenching efficiency than those pretreated with 0 or 200 mM ZIL. These results show that ZIL pretreatment enhanced the cell wall permeability at 400 mM, where it can dissolve microcrystalline cellulose (Figure 2C and D) and reduce the density of cellulose microfibrils present in the cell walls (Figure 3A), without serious disruption of the cell wall (Figure S3) or significant cytotoxicity (Figure 2B).

### Enhanced Nuclear Transfection Efficiency of CPP‐MC by ZIL Pretreatment

**Figure 4:**
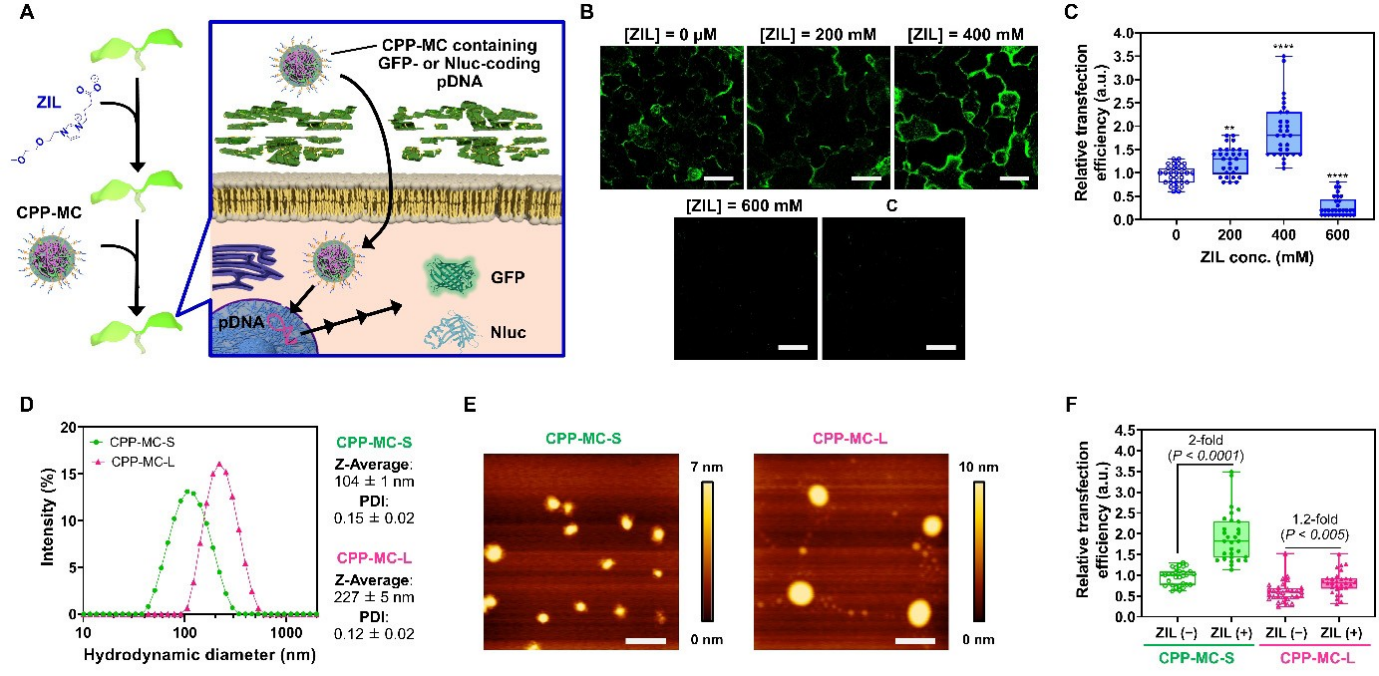
Effects of ZIL on the nuclear transfection efficiency of CPP‐MC in plants. A) Schematic illustration of CPP‐MC‐mediated reporter gene (GFP or NanoLuc^TM^ luciferase (Nluc)) transfection of the nucleus in ZIL‐pretreated plants. B) CLSM images showing GFP expression in epidermal cells in ZIL‐pretreated *A. thaliana* cotyledons 24 h after transfection with CPP‐MC or naked pDNA. C, control sample transfected with the naked pDNA. Scale bars, 40 μm. Chlorophyll fluorescence and bright‐field images corresponding to the GFP fluorescent images are shown in Figure S7. C) Boxplot representation of the relative transfection efficiency of CPP‐MC based on the Nluc expression levels in ZIL‐untreated and ZIL‐pretreated *A. thaliana* seedlings 24 h post‐infiltration: the boxes represent the interquartile range, the lines within the boxes represent the median values, and the upper and lower whiskers represent the highest and lowest values, respectively. Statistical significance compared to the control (ZIL conc., 0 mM): ** *P*<0.01, **** *P*<0.0001 based on Dunnett's T3 test (*n*=30 biological replicates). D) Intensity size distributions, Z‐average diameters, and PDI of CPP‐MC‐S and CPP‐MC‐L based on DLS measurements (*n*=3). E) AFM height images of CPP‐MC‐S and CPP‐MC‐L. Scale bars, 200 nm. Color bars represent the height of the micelle. F) Boxplot representation of the relative transfection efficiency of CPP‐MC‐S and CPP‐MC‐L based on the Nluc expression levels in ZIL‐untreated and ZIL‐pretreated *A. thaliana* seedlings 24 h post‐infiltration. Statistical significance between the ZIL‐untreated and ZIL‐pretreated samples for each micelle was determined by Dunnett's T3 test (*n*=30 biological replicates).

We aimed to explore the effect of ZIL pretreatment on the nuclear transfection of living plants mediated by CPP‐MC (Figure 4A). To this end, we prepared CPP‐MC containing a reporter gene (GFP or NanoLuc^TM^ luciferase (Nluc))‐coded pDNA based on our previous work.^[4g]^ Briefly, we mixed maleimide‐containing polycation peptide (MAL‐TEG‐(KH)_14_) and the reporter gene‐coded pDNA to obtain a maleimide‐presenting micelle complex. Through thiol‐maleimide click chemistry, the surface of the micelle complex was modified with a dual‐domain CPP (CKXAKXAKXAGWWG‐NH_2_, X=α‐aminoisobutyric acid (Aib)), composed of dual cell‐penetrating and endosome‐disrupting domains,^[19]^ to yield CPP‐MC (Figure S4A). We have previously shown that the cell‐penetrating domain contributes to the cellular uptake of micelles via endocytosis while endosome‐disrupting one enhances the cytosolic translocation of micelles from endosomes.^[4g]^ Almost all maleimide groups (95 %) on the micelle surface were conjugated to the dual‐domain CPP based on high‐performance liquid chromatography (HPLC) and matrix‐assisted laser desorption/ionization time‐of‐flight mass spectrometry (MALDI‐TOF MS) analyses (Figure S4). Both CPP‐MCs containing GFP‐ and Nluc‐encoded pDNA were positively charged spherical particles (approximately 100 nm in diameter) with a narrow size distribution, according to dynamic light scattering (DLS), zeta potential, and AFM analyses (Figure S4).

We confirmed that CPP‐MC displaying the dual‐domain CPP transfected *A. thaliana* seedlings more efficiently than did a previously reported endosome‐escaping micelle system that separately displays cell‐penetrating and endosome‐disrupting domains (Figure S5). By the vacuum/compression method, which assisted the transport of nanocarriers into the intercellular space through stomata, hydathodes, and root hairs,^[20]^ CPP‐MC containing GFP‐coded pDNA was infiltrated into whole *A. thaliana* seedlings pretreated with various concentrations of ZIL for 3 h. Note that seedlings were rinsed with water before CPP‐MC‐mediated transfection, because the high concentrations of ZIL (>400 mM) caused aggregation of CPP‐MC (Figure S6). We observed GFP gene expression in the cotyledons pretreated with various concentrations of ZIL followed by transfection with CPP‐MC using time‐gated CLSM imaging,^[21]^ which eliminated the autofluorescence of chlorophyll from the GFP channel. CLSM observations at 24 h post‐transfection indicated GFP expression in the cotyledons pretreated with 0, 200, and 400 mM ZIL followed by CPP‐MC‐mediated transfection (Figures 4B and S7). In contrast, no GFP expression was detected in the cotyledons pretreated with 600 mM ZIL followed by transfection with CPP‐MC and those transfected with naked pDNA (control samples). The GFP gene expression lasted for 48 h after transfection (Figure S8), but no expression was detected at 72 h post‐transfection. In addition to the cotyledons, GFP expression was successfully confirmed in the roots (Figure S9). GFP expression in the transfected seedlings was further supported by western blot and reverse transcription‐quantitative polymerase chain reaction (RT‐qPCR) analyses (Figure S10).

To quantitatively evaluate the transfection efficiency, the seedlings were transfected with CPP‐MC containing Nluc‐coded pDNA after pretreatment with various concentrations of ZIL. We determined the transfection efficiency based on Nluc expression at 24 h post‐transfection, where the maximum expression was observed for ZIL‐untreated seedlings (Figure S11). The Nluc‐based transfection efficiency was significantly enhanced by pretreatment with 200 and 400 mM ZIL compared with the control (0 mM ZIL) (Figure 4C). However, the degree of enhancement was greater at 400 mM than at 200 mM, which is in line with the results obtained from WAXD (Figure 2C), AFM (Figure 2D), and CLSM analyses (Figure 3). Similar to the results of GFP transfection (Figure 4B), pretreatment with 600 mM ZIL markedly reduced the Nluc‐based transfection efficiency (Figure 4C), most likely because of its cytotoxicity (Figure 2B).

As CPP‐MC can be used for transfection of different organ systems, we compared the transfection efficiency of CPP‐MC between shoots and roots from *A. thaliana* seedlings based on the Nluc gene expression level. No significant difference in the transfection efficiency was observed between the shoot and root, while ZIL pretreatment enhanced CPP‐MC‐mediated transfection in both organ systems (Figure S12). These results show that our approach using ZIL and CPP‐MC can be applied to both organ systems.

We further tested the effect of ZIL pretreatment on nuclear transfection mediated by two different sized CPP‐MCs, referred to as CPP‐MC‐S and CPP‐MC‐L. DLS measurements revealed that CPP‐MC‐S (prepared in 5 mM HEPES buffer) was 104 nm in diameter, while CPP‐MC‐L (prepared in 20 mM HEPES buffer) was 227 nm in diameter (Figure 4D). The AFM height images showed a similar trend: spherical particles with smaller diameters were observed for CPP‐MC‐S, and those with larger diameters were detected for CPP‐MC‐L (Figure 4E), even though the height of the particles appeared to decrease upon drying. The zeta potential and CPP modification rate were similar between CPP‐MC‐S and CPP‐MC‐L (Figure S4).

We determined the Nluc‐based transfection efficiency of CPP‐MCs in ZIL‐pretreated and untreated seedlings. ZIL pretreatment enhanced the transfection efficiency of CPP‐MC‐S by approximately 2‐fold but produced only a slight (1.2‐fold) increase in the efficiency of CPP‐MC‐L (Figure 4F). The results may suggest two possibilities. One is that ZIL at 400 mM appeared to partially, but not entirely, disrupt the cell wall (Figures 3A and S3), which could allow the efficient cell wall permeation of relatively small particles (≈100 nm), but not large particles (>200 nm). The other is that large particles (>200 nm) could enter plant cells across the plasma membranes less efficiently than small particles (≈100 nm), even if the former was allowed to translocate across the ZIL‐pretreated cell walls. Nanoparticles including our micelle complex system are considered to enter plant cells via endocytosis, especially via clathrin‐mediated endocytosis (CME).^[22]^ However, large particles (>200 nm) may not be suitable for CME‐mediated cellular uptake because their diameter exceeds that of plant clathrin‐coated vesicles (<100 nm),^[23]^ which mediate the intracellular transport of cargo during CME. Further studies are warranted to explore this issue arising from our results.

Agricultural surfactants such as Silwet L‐77 have been shown to enhance plant transfection by improving the wetting of plant surfaces.^[24]^ We compared the effect of Silwet L‐77 on CPP‐MC‐mediated transfection with that of ZIL. *A. thaliana* seedlings were pretreated for 3 h with ZIL (400 mM) or Silwet L‐77 (0.005 % or 0.05 %, v/v, previously reported concentrations for plant transformation),^[24]^ followed by infiltration with CPP‐MC. The Nluc‐based transfection efficiency of CPP‐MC was 2.1‐fold higher in the ZIL‐pretreated seedlings than that in the Silwet L‐77 (0.05 %, v/v)‐pretreated ones (Figure S13). Pretreatment with the higher concentration (0.05 %, v/v) of Silwet L‐77 resulted in the markedly decreased transfection efficiency (Figure S13), likely due to the cytotoxic effects. These results highlight the superiority of ZIL to Silwet L‐77 in nanocarrier‐mediated pDNA delivery to plants and may be explained by the different modes of action between ZIL and Silwet L‐77. ZIL can enhance the permeability of plant cell walls by disrupting hydrogen bonds between the cell wall components, allowing efficient translocation of nanocarriers across the cell wall. In contrast, Silwet L‐77 can help transport nanocarriers into intracellular spaces by improving the wetting of plant surfaces,^[6]^ but it might not boost the cell wall translocation of nanocarriers.

We extended our method using ZIL and CPP‐MC to transfection of mature plants. Ten‐week‐old *A. thaliana* leaves were subjected to ZIL pretreatment followed by CPP‐MC‐mediated transfection. According to the expression of reporter genes (GFP and Nluc), CPP‐MC was successfully used for the transfection of mature leaves of *A. thaliana* (Figure S14A), and the transfection efficiency was enhanced by 2.4‐fold in the ZIL pretreatment (Figure S14B), similar to the results obtained from young seedlings. These results show that our method can be applied to mature plants as well as young seedlings.

### Enhanced Chloroplast Transfection Efficiency of CTP/CPP‐MC by ZIL Pretreatment

**Figure 5:**
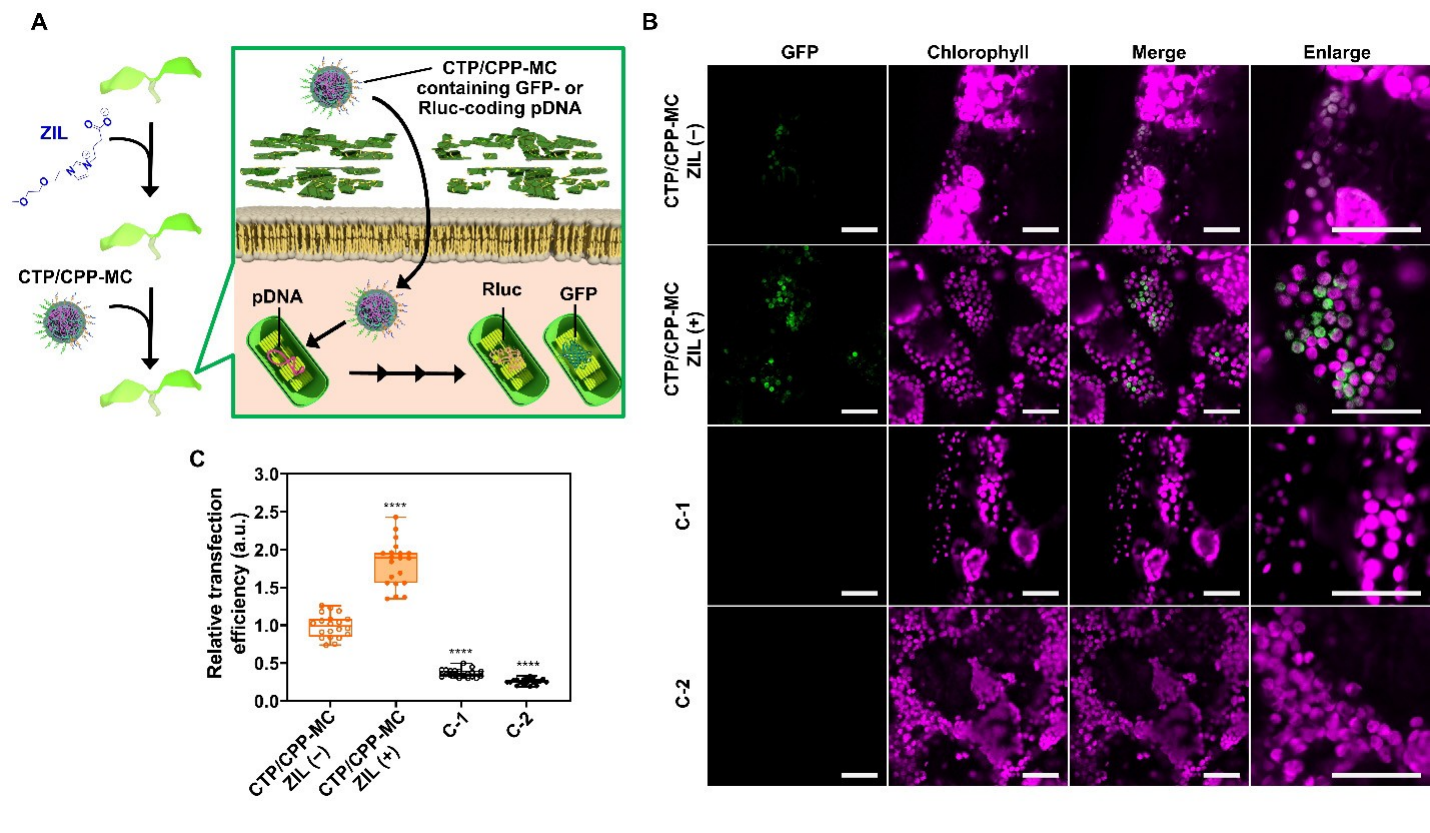
Effects of ZIL on the chloroplast transfection efficiency of CTP/CPP‐MC in plants. A) Schematic illustration of CTP/CPP‐MC‐mediated reporter gene (GFP or *Renilla* luciferase (Rluc)) transfection of chloroplasts in ZIL‐pretreated plants. B) CLSM images showing GFP expression in epidermal cells in ZIL‐untreated and ZIL‐pretreated *A. thaliana* cotyledons 24 h after transfection with CTP/CPP‐MC or controls (naked pDNA or CTP/CPP‐MC containing pDNA for nucleus transfection (P35S‐GFP‐Tnos)). Scale bars, 40 μm. C) Boxplot representation of the relative transfection efficiency of each system based on the Rluc expression levels in ZIL‐pretreated *A. thaliana* seedlings 24 h post‐infiltration. Statistical significance compared to the control (CTP/CPP‐MC, ZIL (−)): **** *P*<0.0001 based on Dunnett's T3 test (*n*=20 biological replicates).

The enhanced pDNA transfection into plant nuclei by ZIL pretreatment motivated us to investigate the effect of ZIL on chloroplast‐targeted transfection mediated by CTP/CPP‐MC (Figure 5A). We obtained CTP/CPP‐MC by modifying the MAL‐TEG‐(KH)_14_/pDNA (coding GFP or *Renilla* luciferase (Rluc)) micelle complex with CPP and CTP designed from Rubisco small subunit 1A (MASSMLSSATMVGGC‐NH_2_) (Figure S15A).^[14]^ RP‐HPLC and MALDI‐TOF MS analyses revealed the conjugation of CPP and CTP to ≈45 and ≈50 % of the maleimide groups on the micelle surface, respectively (Figure S15B and S15C). Based on DLS, zeta potential, and AFM analyses (Figure S15C–S15E), CTP/CPP‐MCs containing GFP‐ or *Renilla* luciferase (Rluc)‐encoding pDNA exhibited similar physicochemical properties: approximately 120 nm in diameter, positive surface charge, and spherical shape.

To investigate the subcellular localization of CTP/CPP‐MC, we pretreated *A. thaliana* seedlings with 400 mM ZIL for 3 h followed by infiltration with fluorescently labeled micelle complexes. Before infiltration, ZIL was removed from the seedlings by rinsing with water to avoid the aggregation of the micelle complex (Figure S16). After 12 h of incubation, CLSM observations confirmed that CTP/CPP‐MC was translocated to chloroplasts, unlike CPP‐MC (Figure S17), which was located mainly in the cytosol and partly in the nucleus.

CTP/CPP‐MC containing the GFP‐coded pDNA was transfected into *A. thaliana* seedlings untreated or pretreated with 400 mM ZIL. We observed the GFP expression in the transfected seedlings using time‐gated CLSM imaging.^[21]^ The time‐gated CLSM images clearly indicated GFP expression in the chloroplasts of the transfected seedlings regardless of ZIL pretreatment (Figure 5B). Similar to CPP‐MC‐mediated nuclear transfection (Figure S8), the CTP/CPP‐MC‐mediated GFP expression in chloroplasts lasted up to 48 h post‐transfection (Figure S18). Conversely, no GFP expression was observed in controls (Figure 5B), i.e., the seedlings transfected with the naked pDNA or CTP/CPP‐MC containing the pDNA for GFP expression in the nucleus (P35S‐GFP‐Tnos). A similar trend was also observed in western blot and RT‐qPCR analyses (Figure S19), validating that CTP/CPP‐MC achieved chloroplast‐specific pDNA transfection. Like the Nluc‐based transfection efficiency of CPP‐MC (Figure S11), the Rluc‐based transfection efficiency of CTP/CPP‐MC was higher at 24 h post‐transfection than that at 48 and 72 h post‐transfection (Figure S20). The transfection efficiency of CTP/CPP‐MC doubled after ZIL pretreatment (Figure 5C), which was consistent with the results of CPP‐MC‐mediated nuclear transfection (Figure 4C). From these results, we conclude that our ZIL pretreatment can promote CTP/CPP‐MC‐mediated chloroplast‐specific transfection, as well as CPP‐MC‐mediated nuclear transfection.

To date, ILs including ZIL have been used as a cellulose dissolving solvent for cellulose utilization and biomass processing.^[25]^ This study has extended the use of ZIL to nanocarrier‐mediated pDNA delivery in living plants. We show for the first time that ZIL pretreatment can enhance the transfection efficiency of nanocarriers by improving the permeability of the plant cell wall with negligible cytotoxicity. Nanocarrier‐mediated pDNA delivery to plant organelles is severely hampered by the size exclusion limit of the plant cell wall, but few studies offered an effective strategy to relax this limitation. Our findings can provide a possible clue for overcoming the plant cell wall barrier for bioengineering of plant organelles.

We previously showed that CTP/CPP‐mediated chloroplast transfection was more efficient and selective in several plant species than conventional particle bombardment‐mediated transfection.^[5c]^ In this study, by combining CTP/CPP‐MC and ZIL pretreatment, we further augmented its chloroplast transfection efficiency. Unlike particle bombardment, the use of ZIL and CTP/CPP‐MC can overcome the dual barrier of the cell wall and plasma membrane without strong mechanical aid provided by expensive equipment. As such, our approach could be a promising alternative to particle bombardment. Furthermore, our approach can avoid cumbersome cell wall removal by enzymes, which is necessary for polyethylene glycol‐mediated protoplast transfection. Although we demonstrated the utility of our method in a model dicot plant (*A. thaliana*), our approach intended to disturb the hydrogen bonds of the wall components may be applicable to different plant species, because hydrogen bonding commonly plays an essential role in the formation of a complex network of the cell wall for all land plants. Meanwhile, the composition and structure of plant cell walls can differ among cell types and developmental stages.^[26]^ Our method will have to be optimized in terms of ZIL concentration and pretreatment time for target plant materials, which may lead to widespread applications in plant bioengineering.

## Conclusion

We have presented a unique approach that synergistically employs a cell wall‐disruptive zwitterionic liquid (ZIL) and an organelle‐targeting micelle complex for pDNA delivery to specific plant organelles. Our results demonstrated that ZIL pretreatment under optimal conditions can enhance the permeability of the plant cell wall, most likely via partial dissolution of cellulose, without causing cytotoxicity to the plants because of its compatibility with the plasma membranes. Owing to the ability of ZIL to relax the size exclusion limit of the plant cell wall, the efficiency of micelle‐complex‐mediated transfection into both the nuclei and chloroplasts of a model plant was more than doubled. Notably, unlike polyethylene glycol‐mediated protoplast transfection, ZIL pretreatment does not rely on enzymatic degradation to mitigate the cell wall barrier, thereby avoiding the difficulty of plant regeneration from protoplasts. In addition, the micelle complex enables pDNA delivery to specific plant organelles, such as chloroplasts, without specialized and expensive equipment, which is necessary for particle bombardment. These advantages could make our approach more feasible for widespread applications in plant bioengineering and plant biology studies than the existing methods.

Although the transfected pDNA may not be integrated into the host genome (especially the nuclear genome) in our method, this might be useful for non‐transgenic plant engineering. In this point of view, a possible application of our method could be transgene‐free plant genome editing, in which the micelle complex delivers the pDNA cargo for the transient expression of genome editing nucleases, such as zinc‐finger nucleases (ZFNs), transcription activator‐like effector nucleases (TALENs) and clustered regularly interspaced short palindromic repeat (CRISPR)‐Cas‐associated nucleases. Meanwhile, when a pDNA cargo is designed for homologous recombination and delivered to chloroplasts by our method, the pDNA cargo may be integrated into the chloroplast genome via homologous recombination. Indeed, we previously showed homologous recombination‐mediated pDNA integration into the chloroplast genome in a model plant.^[27]^ By combining with the pDNA cargo for homologous recombination, our method may be applied to chloroplast transformation, even though further studies are needed.

The concept of utilizing a cell wall‐loosening ZIL may be combined with various types of nanomaterials, as long as they are smaller than ≈200 nm, potentially expanding the utility of nanotechnology in many applications to meet the increasing demand for the sustainable production of foods, materials, and energy. Besides the use of ZIL in nanotechnology, the combination of ZIL with the existing plant biotechnologies, such as enzyme‐mediated protoplast generation and particle bombardment‐mediated transformation, may be possible future directions. In summary, this study provides a clue to overcoming the largely impregnable barrier of the plant cell wall for nanocarrier‐mediated organelle‐specific cargo delivery as well as other applications in plant nanobiotechnology.

## Experimental

Experimental details (PDF) are given in the Supporting Information.

## Conflict of interest

The authors declare no conflict of interest.

1

## Supporting information

As a service to our authors and readers, this journal provides supporting information supplied by the authors. Such materials are peer reviewed and may be re‐organized for online delivery, but are not copy‐edited or typeset. Technical support issues arising from supporting information (other than missing files) should be addressed to the authors.

Supporting Information

## Data Availability

The data that support the findings of this study are available in the Supporting Information of this article.
