# Supplementary material for: Relaxation of the Plant Cell Wall Barrier via Zwitterionic Liquid Pretreatment for Micelle‐Complex‐Mediated DNA Delivery to Specific Plant Organelles
Source: Angew Chem Int Ed Engl. 2022 Jun 24;61(32):e202204234. doi: 10.1002/anie.202204234 (PMC9401069; doi:10.1002/anie.202204234)
Supplement: Supplementary file 1 — Supporting Information [file ANIE-61-0-s001.pdf]

Angewandte Chemie
Eine Zeitschrift der Gesellschaft Deutscher Chemiker

Supporting Information

Relaxation of the Plant Cell Wall Barrier via Zwitterionic Liquid Pretreatment for Micelle-Complex-Mediated DNA Delivery to Specific Plant Organelles

T. Miyamoto, K. Tsuchiya, K. Toyooka, Y. Goto, A. Tateishi, K. Numata**

Supporting Information

Contents

Experimental p. S3–S7

Supplementary figures

**Fig. S1**	Effects of ZIL and ILs on plant growth.	p. S8
**Fig. S2**	AFM height images of cellulose microcrystals pretreated with various concentrations of ZIL.	p. S9
**Fig. S3**	Effects of ZIL pretreatment on *A. thaliana* cotyledon cell walls examined by electron microscopy.	p. S10
**Fig. S4**	Characterization of CPP-MC.	p. S11
**Fig. S5**	Comparison of peptide-displaying micelle complexes (MCs) in terms of the transfection efficiency.	p. S12
**Fig. S6**	Effects of ZIL on hydrodynamic diameters of CPP-MC determined by DLS measurements.	p. S13
**Fig. S7**	CLSM images showing GFP expression in epidermal cells in transfected *A. thaliana* cotyledons pretreated with various concentrations of ZIL.	p. S14
**Fig. S8**	Time course evaluation of CPP-MC-mediated nuclear GFP expression in *A. thaliana* cotyledons.	p. S15
**Fig. S9**	CLSM images showing GFP expression in roots 24 h after CPP-MC-mediated transfection with or without ZIL.	p. S16

**Fig. S10**	Validation of CPP-MC-mediated GFP expression in *A. thaliana* seedlings.	p. S17
**Fig. S11**	Time course evaluation of Nluc-based transfection efficiency of CPP-MC in *A. thaliana* cotyledons.	p. S18
**Fig. S12**	Comparison of transfection efficiency of CPP-MC between shoots and roots from *A. thaliana* seedlings with or without ZIL pretreatment.	p. S19
**Fig. S13**	Comparison of CPP-MC-mediated transfection efficiency in ZIL-wand Silwet L-77-pretreated *A. thaliana* seedlings.	p. S20
**Fig. S14**	Effects of ZIL on CPP-MC-mediated transfection in mature plant leaves.	p. S21
**Fig. S15**	Characterization of CTP/CPP-MC.	p. S22
**Fig. S16**	Effects of ZIL on hydrodynamic diameters of CTP/CPP-MC determined by DLS measurements.	p. S23
**Fig. S17**	CLSM images showing the subcellular localization of the fluorescently labeled micelles in *A. thaliana* cotyledons pretreated with 400 mM ZIL.	p. S24
**Fig. S18**	Time course evaluation of CTP/CPP-MC-mediated chloroplast GFP expression in *A. thaliana* cotyledons.	p. S25
**Fig. S19**	Validation of chloroplast-specific GFP expression in the transfected *A. thaliana* seedlings.	p. S26
**Fig. S20**	Time course evaluation of Rluc-based transfection efficiency of CTP/CPP-MC in *A. thaliana* cotyledons.	p. S27

Supplementary references p. S28

Experimental

Peptides, pDNA, and plants

From the Research Resources Division of RIKEN Center for Brain Science, we obtained the following synthetic peptides: CPP (sequence: CKXAKXAKXAGWWG-NH_2, X = α-aminoisobutyric acid (Aib); Mw: 1459) and CTP (sequence: MASSMLSSATMVGGC-NH_2; Mw: 1431). A previously reported peptide, MAL-TEG-$(KH)_{14}$ (sequence: MAL-TEG-KHKHKHKHKHKHKHKHKHKHKHKHKHKH-NH_2; Mw: 4128),[1] was used to prepare micelle complexes. The purity of each peptide was over 95% based on reverse-phase high-performance liquid chromatography (RP-HPLC). The previously described plasmids were used for transfection: P35S-NanoLuc™ luciferase (Nluc)-Tnos and P35S-GFP-Tnos for nuclear transfection,[1] PpsbA-*Renilla* luciferase (Rluc) and Prrn-GFP-TpsbA for chloroplast transfection.[2] Wild-type (Col-0) or transgenic (YFP-expressing) *Arabidopsis thaliana* (a model dicot plant species) seedlings were grown under previously described conditions.[1] Seedlings at 8–10 days after germination were used for the experiments. Mature plants at 10 weeks after germination were also used for transfection experiments.

Synthesis and characterization of zwitterionic liquid (ZIL)

ZIL was synthesized by a modified protocol according to the previous report.[3] To a suspension of sodium hydride (5.46 g, 60 wt% dispersion in oil, 136.6 mmol) in dry THF (30 mL), a solution of imidazole (9.30 g, 136.6 mmol) in THF (30 mL) was added dropwise at 25 °C under a nitrogen atmosphere. The resulting mixture was stirred at 25 °C for 1 h. To this solution, 1-bromo-2-(2-methoxyethoxy)ethane (25.0 g, 136.6 mmol) was added and the mixture was stirred at 55 °C for 36 h. After the reaction, an inorganic precipitate was removed by filtration, and the solution was concentrated by a rotary evaporator. The crude product was purified by vacuum distillation to provide 1-[2-(2-methoxyethyl)ethyl]-1*H*-imidazole (13.8 g, 80.9 mmol). Then, the imidazole derivative was dissolved in acetonitrile (100 mL) and ethyl 4-bromobutyrate (15.8 g, 81.0 mmol) was added. The mixture was stirred at 70 °C for 24 h. After cooled to 25 °C, the mixture was concentrated by a rotary evaporator and dried under a vacuum. The crude bromide salt was dissolved in distilled water and applied to a column filled with an anion exchange resin (Amberlite IRN78). The flow-through was concentrated by a rotary evaporator and dried under a vacuum at 100 °C to provide ZIL. The yield was 21.55 g (61.5%). The chemical structure was confirmed by ^{1}H NMR (DPX400 spectrometer, Bruker, Bremen, Germany) and all the signals were assignable according to the literature.[3] ^{1}H NMR (400 MHz, $CDCl_3$, ppm) δ 10.88 (1H, s), 7.46 (1H, s), 7.17 (1H, s), 4.65 (2H, t, J = 4.6), 4.40 (2H, t, J = 6.8), 3.89 (2H, t, J = 4.4), 3.64 (2H, t, J = 4.4), 3.52 (2H, t, J = 4.4), 3.37 (3H, s), 2.13-2.27 (4H, m).

Cytotoxicity of ZIL to plants

Five seedlings of wild-type *A. thaliana* were immersed in an aqueous solution (200 μL) containing various concentrations (0–800 mM) of ZIL, IL-1 (1-butyl-3-methylimidazolium acetate, Tokyo chemical industry), or IL-2 (1-ethyl-3-methylimidazolium acetate, Sigma-Aldrich) at 25 °C for 3 h. The seedlings were washed with Milli-Q water. After incubation at 22 °C for 24 h on a half-strength Murashige and Skoog (1/2 MS) medium plate, the viability of the seedlings was determined by the previously reported Evans blue assay.[1]

Data were obtained from four biologically independent samples (each sample consisted of five infiltrated seedlings). The seedlings were photographed at 1, 10, and 20 days after treatment.

Evaluation of the ability of ZIL to dissolve cellulose microcrystals by wide-angle X-ray diffraction (WAXD) and atomic force microscopy (AFM) analyses

The synchrotron WAXD measurements of cellulose nanocrystal pellets were performed similarly as previously reported.[4] A cellulose microcrystal dispersion derived from *Halocynthia sp*. (0.2 %, w/v, 200 μL) was washed with Milli-Q water. The microcrystals were collected after centrifuge (9000 rpm, 25 °C, 10 min) and incubated in an aqueous solution (200 μL) containing various concentrations of ZIL (0–400 mM) at 25 °C for 2 h. After centrifuge (9000 rpm, 25 °C, 15 min), the ZIL-treated microcrystals were collected and lyophilized. Then, the lyophilized microcrystals were subjected to WAXD measurements on the BL05XU beamline (SPring-8, Harima, Japan) using X-ray energy of 15 keV (wavelength: 1.0 Å). The sample-to-detector distance for the WAXD measurements was set to 240 mm, and the exposure time for each diffraction was 1 sec. The obtained two-dimensional (2D) diffraction patterns were azimuthally integrated to convert to one-dimensional (1D) WAXD profiles using Fit2D software. The 1D WAXD profiles were subjected to deconvolution for peak separation. The degree of crystallinity of each sample was calculated from the integral ratio of crystalline peaks to an amorphous halo peak.

For AFM observations, the cellulose microcrystal dispersion (20 μL) was dropped on a highly oriented pyrolytic graphite (HOPG) substrate and the excess solution was immediately wiped away. After washing with Milli-Q water and drying under vacuum, the sample was treated with aqueous solutions containing ZIL (20 μL) at various concentrations (0, 200, or 400 mM) on the HOPG at 25 °C for 2 h. After the excess liquid in the droplet was wiped away, the HOPG substrate was washed with Milli-Q water and dried under a vacuum. The ZIL-treated cellulose microcrystals were analyzed by AFM with a Bruker Multimode 8 AFM (Bruker, Santa Barbara, CA) in Peak Force QNM mode with a cantilever of SCANASYST-AIR-HR (Al reflective coating, force constant: 0.4 N m^{-1}, resonance frequency: 130 kHz) at 25 °C under ambient atmosphere.

Visualization of ZIL-treated cellulose microfibrils in the plant cell wall by confocal laser scanning microscopy (CLSM)

A. thaliana seedlings were treated with ZIL at various concentrations (0, 200, or 400 mM) for 3 h and then washed with Milli-Q water. They were stained with calcofluor white (Sigma-Aldrich) aqueous solution (200 μg/mL) for at least 30 min under dark conditions. After intensive wash with Milli-Q water, they were subjected to CLSM using a Leica TCS SP8 confocal microscope (Leica) under HyVolution mode with Ex/Em wavelengths of 405/420–480 nm for calcofluor white.

Evaluation of ZIL effect on the cell wall morphology and density using transmission electron microscopy (TEM) and field emission-electron scanning microscopy (FE-SEM)

A. thaliana seedlings were treated with an aqueous solution (200 μL) containing ZIL (0, 200, or 400 mM)

for 3 h and then washed with Milli-Q water. Cotyledons of the pretreated seedlings were fixed and embedded in an epoxy resin block as previously described.[5] The serial ultrathin sections (70 nm and 100 nm per section for TEM and FE-SEM, respectively) were obtained from the resin blocks and stained with an uranyl acetate solution for 10 min and a lead citrate solution for 1 min. The samples were observed on a JEM 1400 Flash transmission electron microscope (JEOL) at 80 kV or a SU8220 scanning electron microscope (Hitachi) at acceleration voltage of 5.0 kV.

Quantitative evaluation of cell wall permeability by quenching assay

To quantitatively evaluate cell wall permeability, we performed a quenching assay reported by Liu *et al.*[6] The quenching assay was based on CLSM imaging and image analysis. For CLSM imaging, *A. thaliana* cotyledons were treated with ZIL at various concentrations (0, 200, or 400 mM) for 3 h, washed with 1/2MS medium containing 1% sucrose, and then stained with FM4-64 (50 μM) for 3 min under dark conditions. After washing with 1/2MS medium containing 1% sucrose, cotyledons were further treated for 3 min with various concentrations (0–10 μM) of trypan blue (TB, Sigma-Aldrich) aqueous solutions in 1.5 mL-tube under dark conditions. Immediately after TB treatment, the cotyledon was placed on a grass plate with 50 μL of TB solution (the same concentration as the "3 min-treatment") and imaged using an LSM700 microscope (Carl Zeiss) at Ex/Em wavelengths of 488/620–680 nm for FM4-64. The obtained CLSM images were analyzed by Fiji/ImageJ to estimate the quenching efficiency. The FM4-64 fluorescence intensities in the apical plasma membrane regions, which were manually segmented using the polygonal selection tool of Fiji/ImageJ, were calculated for more than eighty regions of interest (ROI) from four biological replicates for each system. For background correction, the fluorescence intensity, obtained from an area where no cells were present, was subtracted from the FM4-64 fluorescence intensity. The quenching efficiency was calculated according to the Stern-Volmer equation, $F_0/F = 1 + K[Q]$, where F_0 and F are the fluorescence intensity of FM4-64 in the absence and presence of TB at the concentration Q, respectively, and K is the quenching coefficient. The quenching efficiency represented by the K value is considered as a quantitative indicator of cell wall permeability.

Preparation and characterization of peptide-displaying micelle complexes

The CPP-modified micelle complex (CPP-MC) was prepared as previously reported.[1] Briefly, MAL-TEG-$(KH)_{14}$ (final concentration, 5.5 μM) and pDNA (final concentration, 25 μg/mL) were mixed in Milli-Q water and incubated for 15 min to obtain an unmodified micelle complex (UM-MC). CPP (final concentration, 6 μM) and HEPES (final concentration, 5 mM, pH 7.4) were added to the solution containing UM-MC and incubated at 25 °C for 30 min to obtain CPP-MC. Using a similar procedure for CPP-MC, we prepared the micelle complex dually modified with CTP and CPP (CTP/CPP-MC). Specifically, UM-MC was incubated with CTP (final concentration, 3 μM) and CPP (final concentration, 3 μM) for 30 min in HEPES buffer (5 mM, pH 7.4) to obtain CTP/CPP-MC. CPP-MC-L was prepared by the surface modification of UM-MC with CPP (final concentration, 6 μM) in 20 mM HEPES (pH 7.4). The reaction mixture containing CPP-MC or CTP/CPP-MC was directly used for characterization and transfection

experiments. CPP-MC and CTP/CPP-MC were characterized through MALDI-TOF MS, RP-HPLC, DLS, zeta potential, and AFM analyses as previously reported.[1] To evaluate the micelle stability, DLS measurements of CPP-MC and CTP/CPP-MC were performed in the presence of various concentrations (0–600 mM) ZIL.

CPP-MC-mediated nucleus transfection in ZIL-treated plants

Seedlings of wild-type *A. thaliana* were treated with various concentrations (0–600 mM) of ZIL for 3 h and washed with Milli-Q water. Using the ZIL-treated seedlings and CPP-MC, we performed transfection experiments as previously reported.[1] Briefly, CPP-MC prepared from GFP- or luciferase-coding pDNA (P35S-GFP-Tnos or P35S-Nluc-Tnos) was infiltrated into the ZIL-treated seedlings by the vacuum/compression method. After 24, 48, and 72 h of incubation on a 1/2 MS medium plate (at 22 °C), GFP expression in the cotyledons or roots was confirmed by CLSM observation with a Leica TCS SP8 microscope (Leica). Ex/Em wavelengths of 488/500–570 nm with time-gating (gate-on time: 0.3–12 ns) were used to detect GFP fluorescence with the elimination of autofluorescence, whereas those of 488/650–710 were employed to visualize chlorophyll in chloroplasts. The luciferase expression level in the seedlings was quantified by the previously reported luciferase assay at 24, 48, and 72 h post-infiltration.[7] For luciferase assay, data were obtained from thirty biologically independent samples (one sample consisted of 10 transfected seedlings) for each system.

Using the same method, leaves from10-week-old *A. thaliana* plants were pretreated with ZIL, infiltrated with CPP-MC, and then subjected to CLSM imaging to detect the GFP expression and luciferase assay (where one sample consisted of 2 transfected leaves) to quantify the luciferase expression level. For comparison between ZIL and Silwet L-77, *A. thaliana* seedlings were pretreated with Silwet L-77 (0.005% or 0.05%, v/v) for 3 h and infiltrated with CPP-MC followed by the luciferase assay.

Subcellular localization of CPP-MC and CTP/CPP-MC in ZIL-treated plants

CPP- or CTP/CPP-MC was prepared from Cy3-labeled pDNA, which was obtained using a Label IT Nucleic Acid Labeling Kit (Mirus Bio), and analyzed by DLS as previously reported.[8] The fluorescently labeled CTP/CPP-MC and CPP-MC were infiltrated into the cotyledon of wild-type and YFP-expressing transgenic *A. thaliana* seedlings (8–9 days after germination), pretreated with 400 mM ZIL for 3 h, respectively. After incubation at 22 °C for 12 h, the cotyledons were washed with Milli-Q water and subjected to CLSM observations with a Leica TCS SP8 microscope (Leica). For the CTP/CPP-MC-infiltrated cotyledons, Ex/Em wavelengths of 520/560–600 nm with time-gating (gate-on time: 0.3–12 ns) were employed to detect Cy3 fluorescence signal with the elimination of autofluorescence from chlorophyll, while those of 520/650–710 nm were utilized to observe chlorophyll in chloroplasts. For the CPP-MC-infiltrated cotyledons, Cy3 and YFP (expressed in the cytosol) fluorescence were detected at Ex/Em wavelengths of 540/570–610 nm and 500/510–530 nm, respectively.

CTP/CPP-MC-mediated chloroplast transfection in ZIL-treated plants

CTP/CPP-MC-mediated chloroplast transfection in the ZIL-treated seedlings was performed similarly to the above-described CPP-MC-mediated transfection. Specifically, the ZIL-treated seedlings were infiltrated with CTP/CPP-MC containing GFP- or luciferase-coding pDNA (Prrn-GFP-TpsbA or PpsbA-Rluc) via the vacuum/compression method, followed by incubation on a 1/2 MS medium plate at 22 °C for 24, 48, or 48 h. GFP expression in chloroplasts was observed by CLSM with a Leica TCS SP8 microscope (Leica). Ex/Em wavelengths of 488/500–570 nm with time-gating (gate-on time: 0.3–12 ns) were used to detect GFP fluorescence with the elimination of autofluorescence from chlorophyll, whereas those of 488/650–710 were employed to visualize chlorophyll in chloroplasts. The luciferase expression level in the seedlings was quantified by the previously reported luciferase assay. For luciferase assay, data were obtained from twenty biologically independent samples (one sample consisted of ten transfected seedlings) for each system.

Evaluation of GFP gene expression in transfected plants by western blot and reverse transcription-quantitative polymerase chain reaction (RT-qPCR) analyses

A. thaliana seedlings were pretreated for 3 h with ZIL (0 or 400 mM), infiltrated with CPP-MC (containing P35S-GFP-Tnos), CTP/CPP-MC (containing Prrn-GFP-TpsbA or P35S-GFP-Tnos) or the naked pDNA by the vacuum/compression method. After incubated for 24 h at 22 °C, ~150 and 12 seedlings were homogenized with a multi-beads shocker (Yasuikikai) in phosphate buffered saline (pH 7.4) containing Protease Inhibitor Cocktail (cOmplete™, Merck) for western blot and RT-qPCR, respectively.

For western blot, the lysate was centrifuged (13,500 rpm, 4 °C, 5 min) and the supernatant was lyophilized. The obtained powder was dissolved in 350 μL of 4 × Laemmli buffer (Bio-Rad Laboratory) containing 2-mercaptoethanol (5%, v/v, Bio-Rad Laboratory) and boiled at 100 °C for 15 min followed by centrifuge (14,000 rpm, 25 °C, 10 min). The supernatant (20 μL) was used for SDS-PAGE and western blot analysis as previously described.[2] Briefly, the GFP band on the membrane was detected using a combination of a rabbit anti-GFP polyclonal antibody (NB600-308, Novus Biologicals) with a horseradish peroxidase (HRP)-conjugated goat antirabbit IgG polyclonal antibody (ab6721, Abcam), and imaged with a Fusion Solo S imaging system (Vilber Lourmat).

For RT-qPCR analysis, RNA was extracted from the lysate and subjected to reverse transcription to synthesis complementary DNA (cDNA) using an RNeasy Plant Mini Kit (Qiagen) and a ReverTra Ace® qPCR RT Master Mix with gDNA Remover (Toyobo), respectively, in accordance with the manufacturer's protocol. The obtained cDNA was used for qRT-PCR using SYBR Green RealTime PCR Master Mix Plus (Toyobo) with gene-specific primers for GFP (forward: 5'-CACTGCCGACAAGCAGAAGAAC-3'; reverse: 5'-CTTCTCGTTGGGGTCTTTGCTCAG-3'). *A. thaliana* ACTIN2 (AtACT2) was used as constitutive housekeeping gene expression controls in qRT-PCR with the primers (forward: 5'-TGTGCCAATCTACGAGGGTT-3'; reverse: 5'- TTTCCCGCTCTGCTGTTGTG-3'). Expression data were analyzed with the comparative CT ($2^{-\Delta\Delta CT}$) method.

Statistical analysis

Statistical tests and graphs were generated with GraphPad Prism 9.

Supplementary figures

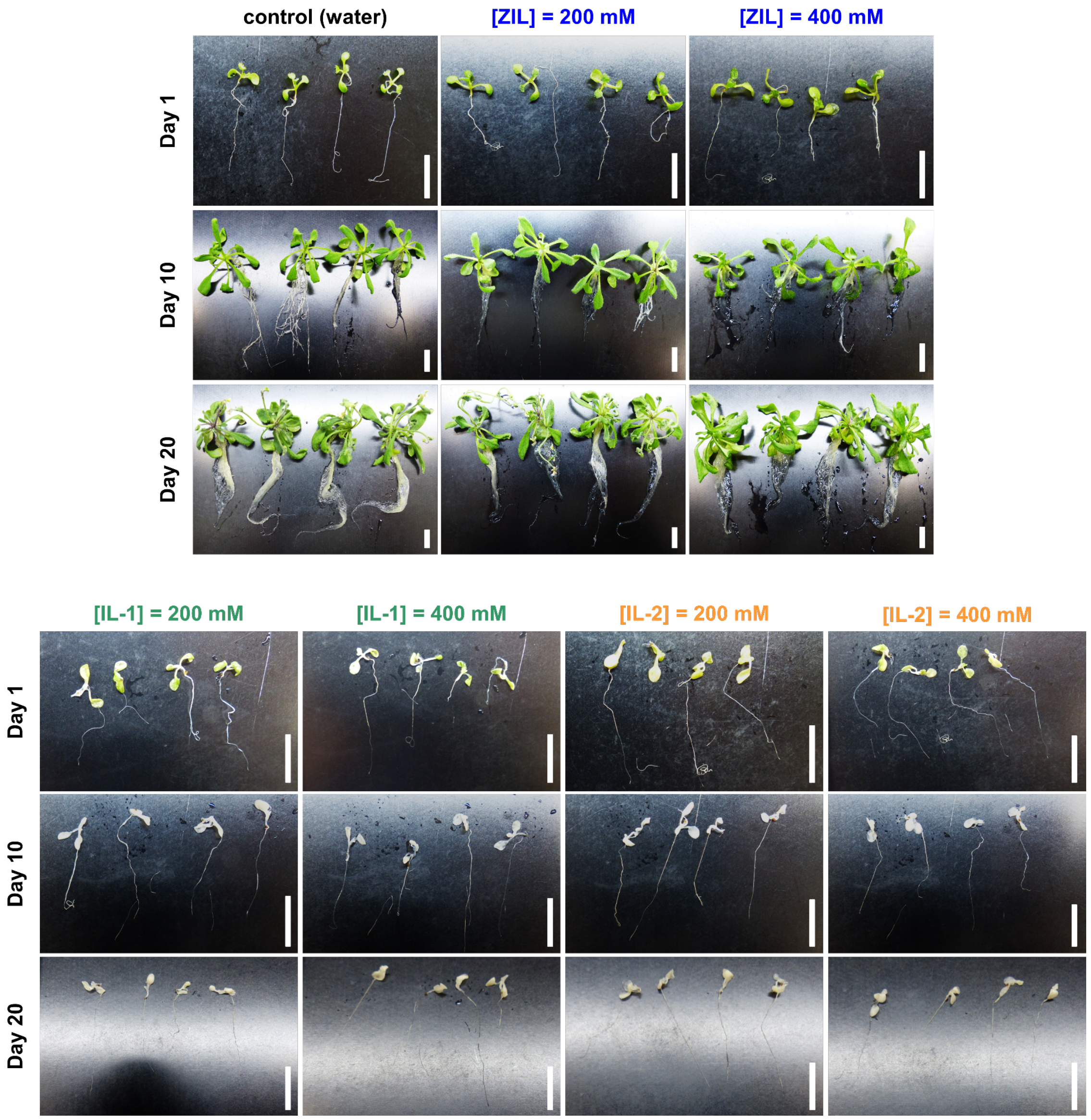

Figure S1. Effects of ZIL and ILs on plant growth. Pictures of *A. thaliana* seedlings at 1, 10, and 20 days after pretreatment with ZIL, IL-1, IL-2, and water for 3 h. Scale bar, 10 mm.

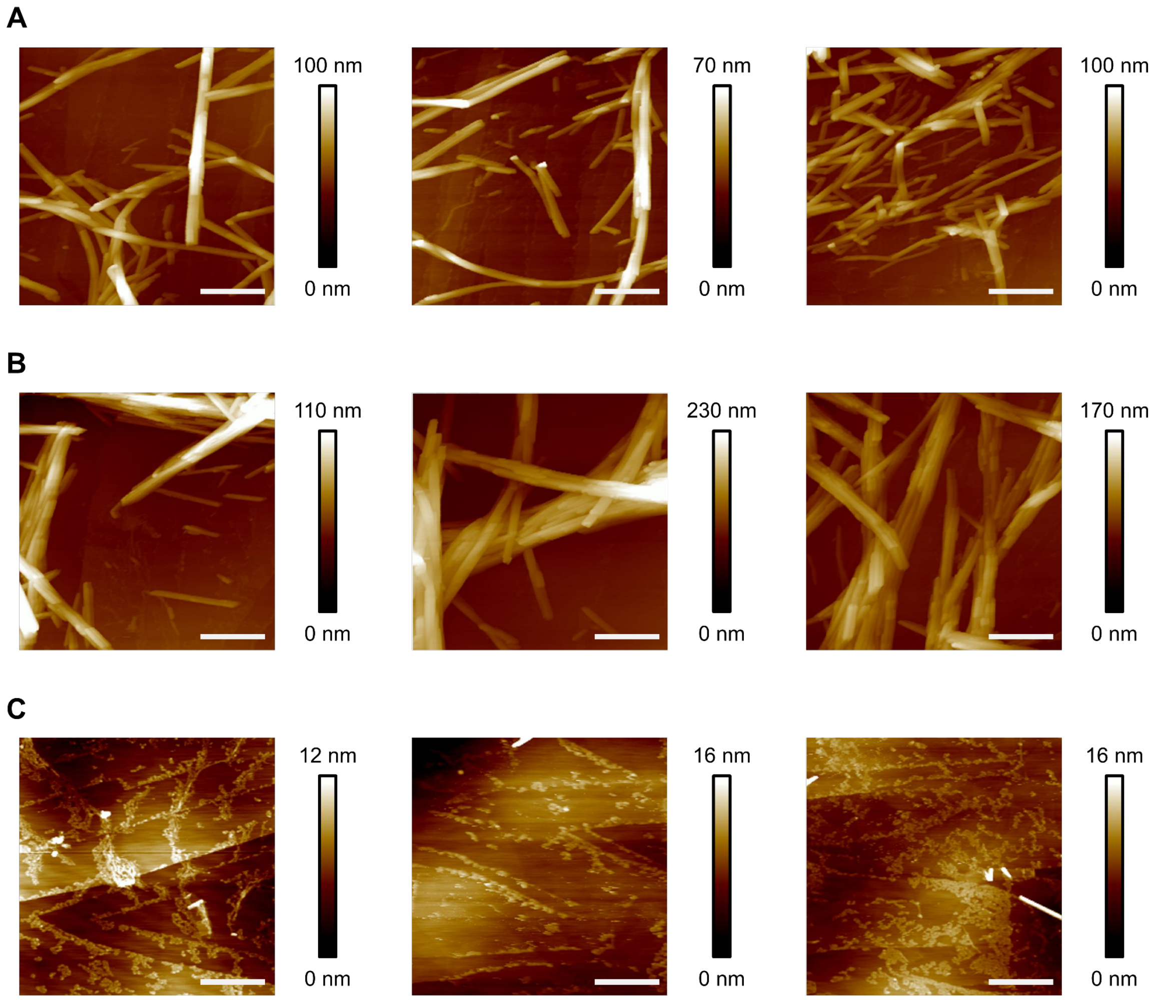

Figure S2. AFM height images of cellulose microcrystals pretreated with various concentrations of ZIL. Morphologies of cellulose microcrystals pretreated for 3 h with (A) 0, (B) 200, and (C) 400 mM ZIL on a HOPG substrate. White bar represents 500 nm and color bars represent the height of the microcrystals.

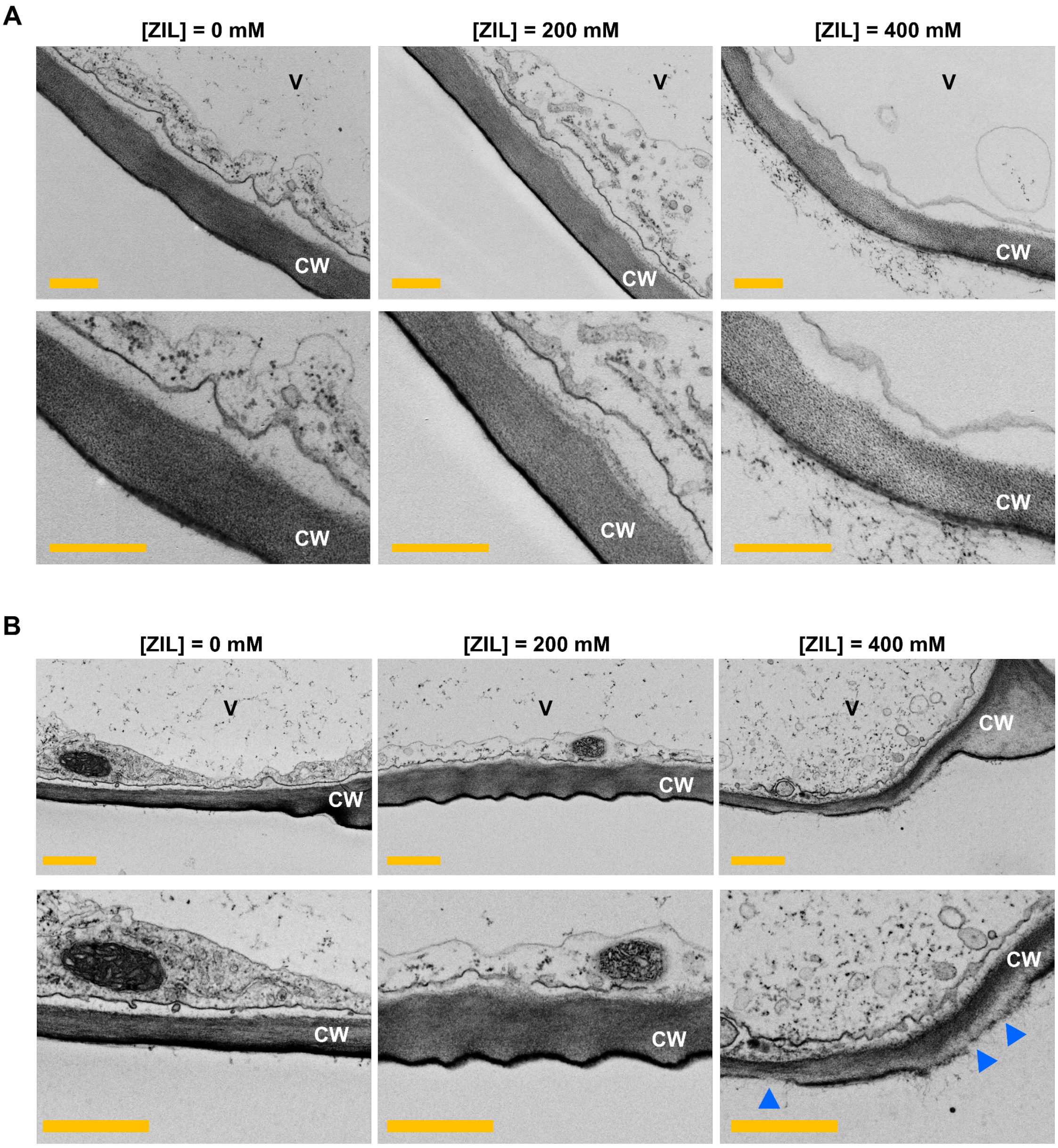

Figure S3. Effects of ZIL pretreatment on *A. thaliana* cotyledon cell walls examined by electron microscopy. (A) TEM and (B) FE-SEM images of the cell walls of epidermal cells in cotyledons after pretreatment with 0, 200, or 400 mM ZIL for 3 h. CW, cell wall. V, vacuole. Scale bars, 0.5 µm. Blue arrows in (B) show loosened cuticle.

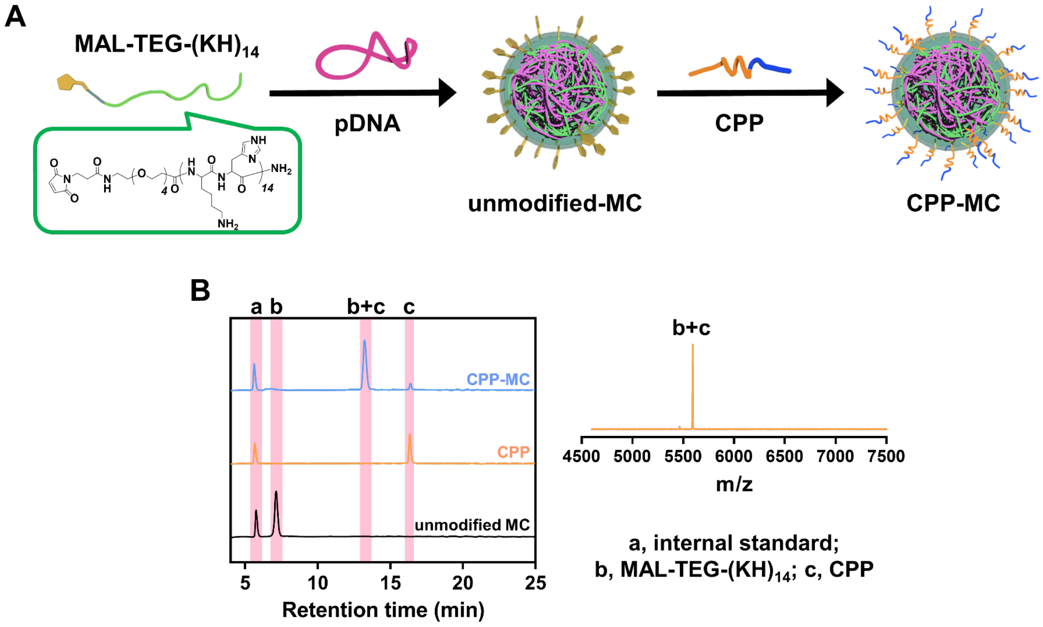

C

	Z-Average (nm)	PDI	Zeta potential (mV)	MAL conv. (CPP, %)
CPP-MC (P35S-GFP-Tnos)	99 ± 1	0.18 ± 0.02	35 ± 1	97 ± 1
CPP-MC-S (P35S-Nluc-Tnos)	104 ± 1	0.15 ± 0.02	29 ± 0	95 ± 0
CPP-MC-L (P35S-Nluc-Tnos)	227 ± 5	0.12 ± 0.02	29 ± 1	94 ± 2

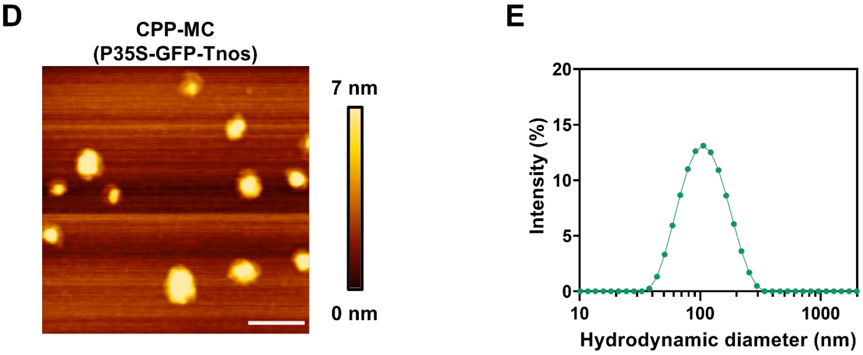

Figure S4. Characterization of CPP-MC. (A) Schematic representation of formation of CPP-MC. (B) RP-HPLC chromatograms of CPP-MC, CPP, and unmodified MC, and MALDI-TOF MS spectrum of the fraction "b+c" obtained from the RP-HPLC. The observed molecular weights of the fractions "b+c" corresponded to the calculated molecular weights of the CPP-MAL-TEG-$(KH)_{14}$ conjugate. (C) Z-average, polydispersity index (PDI), zeta potential, and MAL conversion ratio of GFP-coded pDNA-containing CPP-MC and Nluc-coded pDNA-containing CPP-MC-S and -L. Data are represented as mean ± standard error values (n = 3). (D) AFM height images of GFP-coded pDNA-containing CPP-MC. White bar represents 200 nm and color bars represent the height of the microcrystals. (E) Intensity size distributions of GFP-coded pDNA-containing CPP-MC.

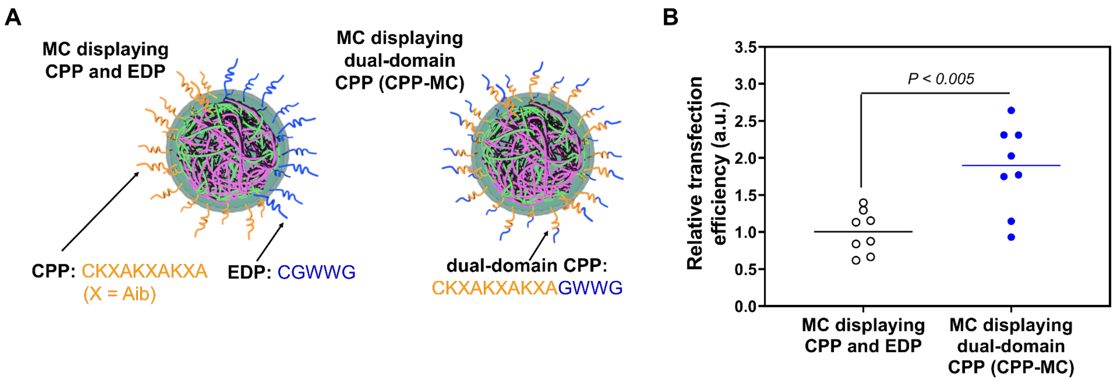

Figure S5. Comparison of peptide-displaying micelle complexes (MCs) in terms of the transfection efficiency. (A) Schematic representation of peptide-displaying MCs. (B) The relative transfection efficiency of peptide-displaying MCs based on Nluc expression levels in *A. thaliana* seedlings 24 h post-infiltration. Statistical significance between two different MCs was determined by Welch's t-test (n = 8 biological replicates). The MC displaying CPP and EDP was prepared as previously reported.[1]

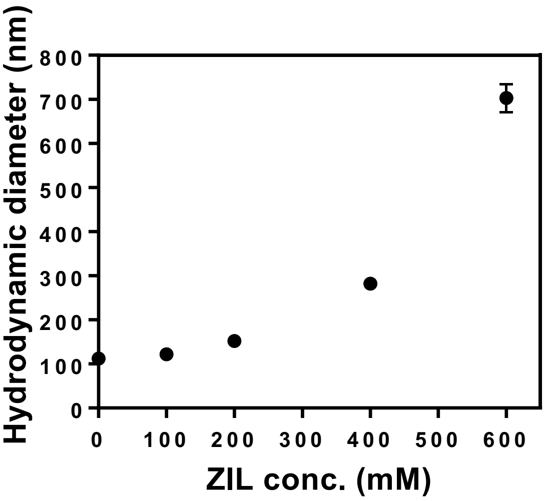

Figure S6. Effects of ZIL on hydrodynamic diameters of CPP-MC determined by DLS measurements. Data from three measurements are represented as the mean ± standard error values.

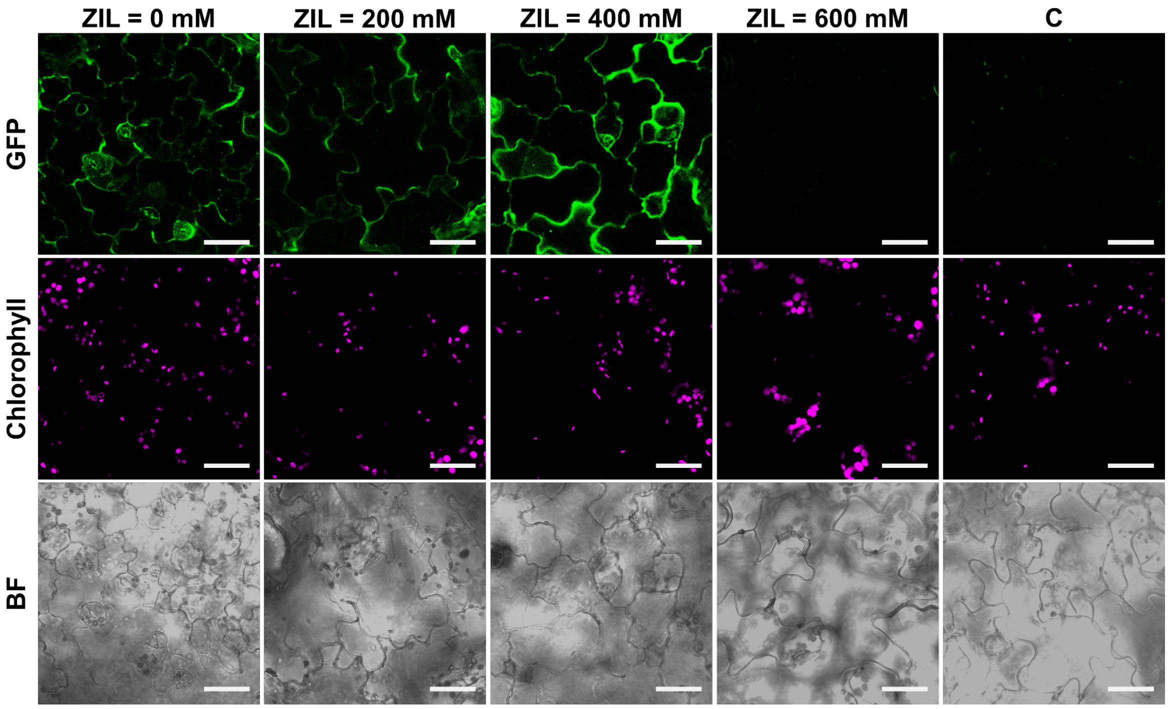

Figure S7. CLSM images showing GFP expression in epidermal cells in transfected *A. thaliana* cotyledons pretreated with various concentrations of ZIL. The CLSM images were obtained at 24 h after transfection with CPP-MC or naked pDNA. C, control sample transfected with the naked pDNA. BF, bright-field image. Scale bars, 40 μm.

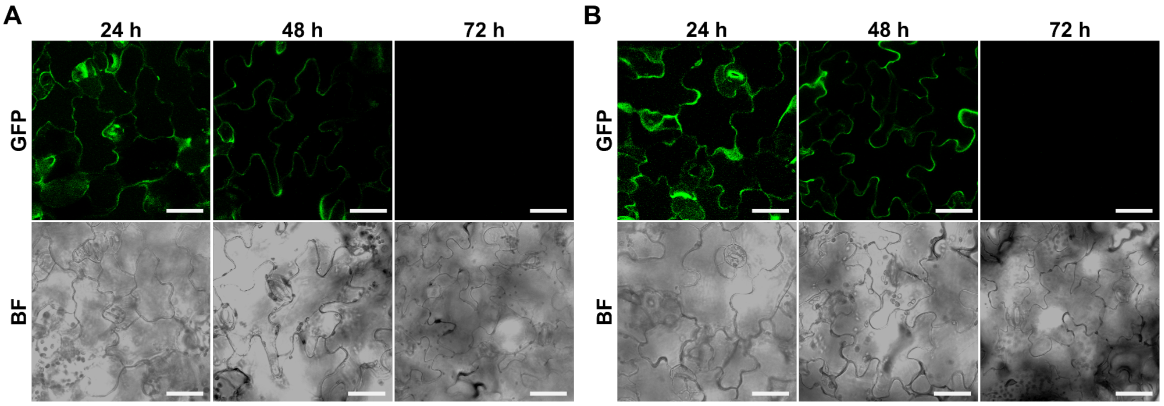

Figure S8. Time course evaluation of CPP-MC-mediated nuclear GFP expression in *A. thaliana* cotyledons. CLSM images obtained from (A) ZIL-untreated and (B) -pretreated samples. BF, bright field. Scale bars, 40 μm.

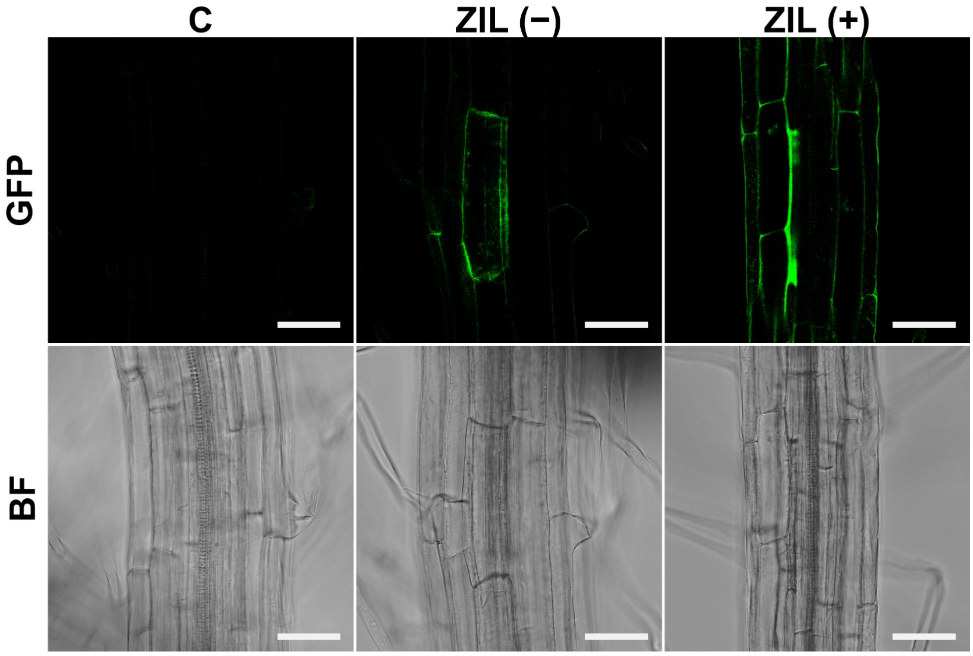

Figure S9. CLSM images showing GFP expression in roots 24 h after CPP-MC-mediated transfection with or without ZIL. C, control sample transfected with the naked pDNA (P35S-GFP-Tnos). ZIL (−), CPP-MC-transfected samples without ZIL pretreatment. ZIL (+), CPP-MC-transfected samples with ZIL pretreatment. Scale bars, 40 μm.

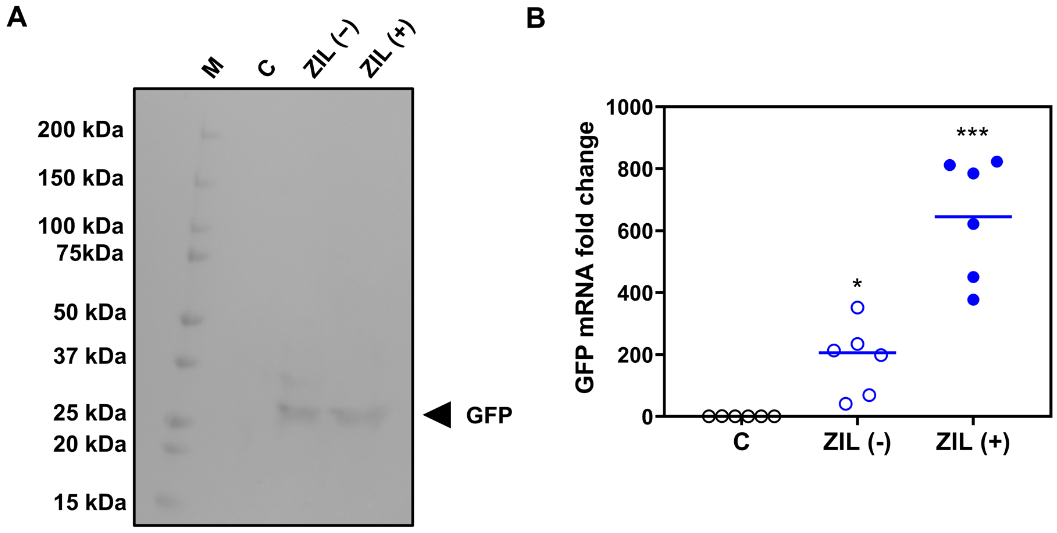

Figure S10. Validation of CPP-MC-mediated GFP expression in *A. thaliana* seedlings. (A) Western blot and (B) RT-qPCR analyses of transfected samples under several conditions. C, control sample transfected with naked pDNA (P35S-GFP-Tnos). ZIL (−), samples untreated with ZIL followed by transfection with CPP-MC containing P35S-GFP-Tnos. ZIL (+), samples pretreated with ZIL followed by transfection with CPP-MC containing P35S-GFP-Tnos. M in (A), molecular weight marker. Statistical significance in (B) was determined by Dunnett's T3 test (n = 6 biological replicates).

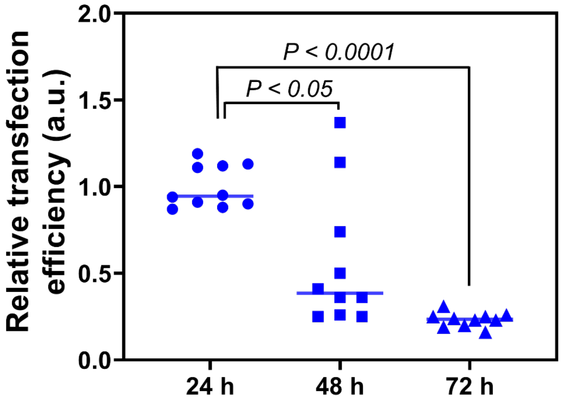

Figure S11. Time course evaluation of Nluc-based transfection efficiency of CPP-MC in *A. thaliana* cotyledons. Statistical significance was determined by Dunnett's T3 test (n = 10 biological replicates).

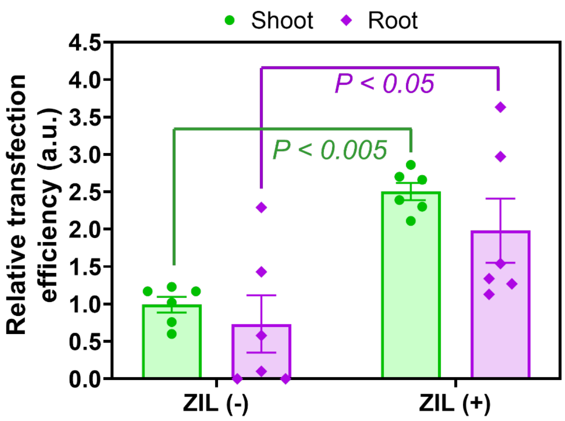

Figure S12. Comparison of transfection efficiency of CPP-MC between shoots and roots from *A. thaliana* seedlings with or without ZIL pretreatment. Data from six biological replicates are represented as the mean ± standard error values. Statistical significance between the ZIL-untreated (ZIL (−)) and ZIL-pretreated (ZIL (+)) samples was determined by Šídák's multiple comparisons test (n = 6 biological replicates).

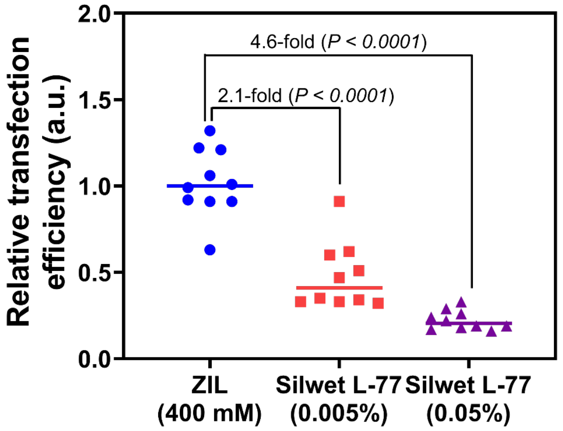

Figure S13. Comparison of CPP-MC-mediated transfection efficiency in ZIL- wand Silwet L-77-pretreated *A. thaliana* seedlings. The relative transfection efficiency of CPP-MC was quantitatively evaluated based on the Nluc expression levels 24 h post-infiltration. Statistical significance between the ZIL- and Silwet L-77-pretreated samples was determined by Dunnett's T3 test (n = 10 biological replicates).

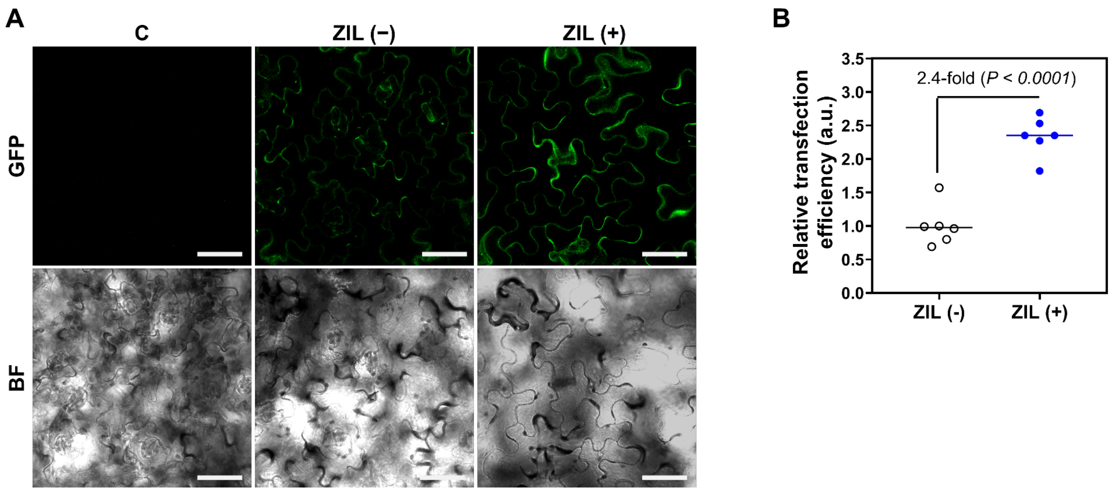

Figure S14. Effects of ZIL on CPP-MC-mediated transfection in mature plant leaves. (A) CLSM images showing GFP expression in ZIL-untreated (ZIL (−)) and -pretreated (ZIL (+)) ten-week-old *A. thaliana* leaves 24 h after transfection with CPP-MC. C, control samples transfected with the naked pDNA. Scale bars, 40 μm. (B) The relative transfection efficiency of CPP-MC based on Nluc expression levels in ten-week-old *A. thaliana* leaves 24 h post-infiltration. Statistical significance between ZIL (−) and ZIL (+) samples was determined by Welch's t-test (n = 6 biological replicates).

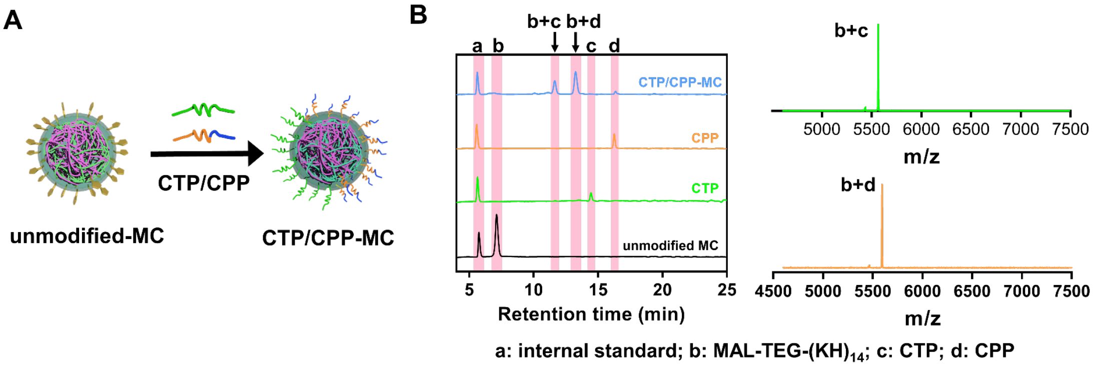

C

	Z-Average (nm)	PDI	Zeta potential (mV)	MAL conv. (CTP, %)	MAL conv. (CPP, %)
CTP/CPP-MC (PpsbA-Rluc)	119 ± 3	0.12 ± 0.01	29 ± 1	44 ± 1	50 ± 0
CTP/CPP-MC (Prrn-GFP-TpbsA)	121 ± 1	0.18 ± 0.01	28 ± 0	46 ± 1	49 ± 2
CTP/CPP-MC (P35S-Nluc-Tnos)	104 ± 1	0.19 ± 0.01	29 ± 1	42 ± 3	47 ± 1
CTP/CPP-MC (P35S-GFP-Tnos)	103 ± 3	0.27 ± 0.01	29 ± 0	44 ± 3	51 ± 2

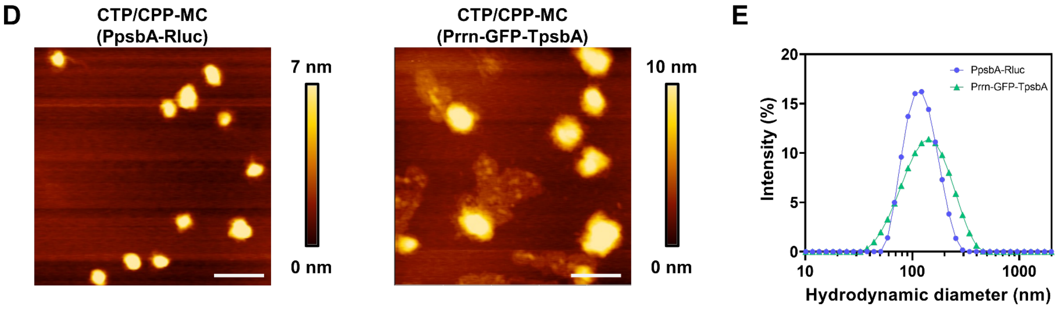

Figure S15. Characterization of CTP/CPP-MC. (A) Schematic representation of formation of CTP/CPP-MC. (B) RP-HPLC chromatogram of CTP/CPP-MC, CPP, CTP, and unmodified MC, and MALDI-TOF MS spectra of the fractions "b+c" obtained from the RP-HPLC. The observed molecular weights of the fractions "b+c" and "b+d" corresponded to the calculated molecular weights of the CTP-MAL-TEG-$(KH)_{14}$ and CPP-MAL-TEG-$(KH)_{14}$ conjugates, respectively. (C) Z-average, polydispersity index (PDI), zeta potential, and MAL conversion ratio of CTP/CPP-MC containing various pDNA: PpsbA-Rluc, Prrn-GFP-TpsbA, P35S-Nluc-Tnos, and P35S-GFP-Tnos. Data are represented as mean ± standard error values (n = 3). (D) AFM height images and (E) intensity size distributions of CTP/CPP-MC containing pDNA of PpsbA-Rluc or Prrn-GFP-TpsbA. White bar represents 200 nm and color bars represent the height of the micelles in (D).

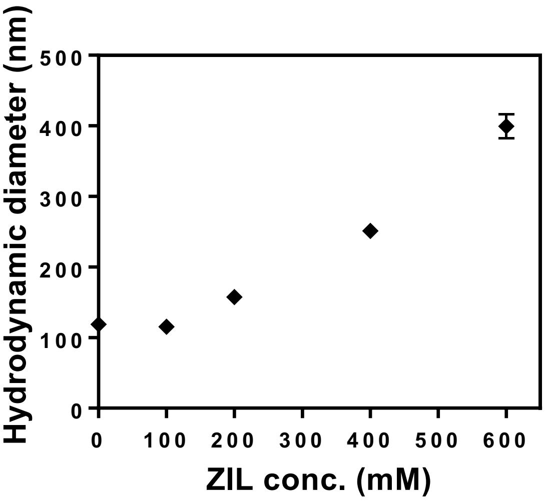

Figure S16. Effects of ZIL on hydrodynamic diameters of CTP/CPP-MC determined by DLS measurements. Data from three measurements are represented as the mean ± standard error values.

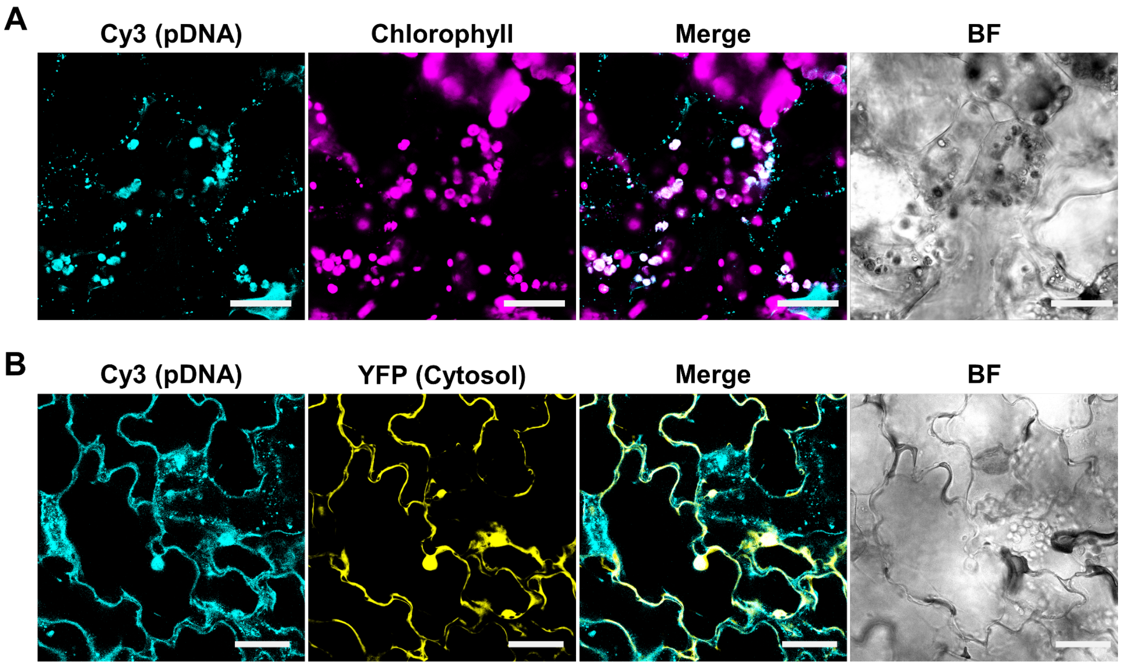

Figure S17. CLSM images showing the subcellular localization of the fluorescently labeled micelles in *A. thaliana* cotyledons pretreated with 400 mM ZIL. The subcellular localization of (A) CTP/CPP-MC in wild-type of cotyledons and (B) CPP-MC in YFP-expressing transgenic cotyledons at 12 h after micelle infiltration. The pDNA, chloroplasts, and cytosol (and nuclei) were visualized by the fluorescence signals of Cy3 (cyan), chlorophyll (magenta), and YFP (yellow), respectively. Epidermal cells are displayed in the "BF" images. Scale bars, 40 µm.

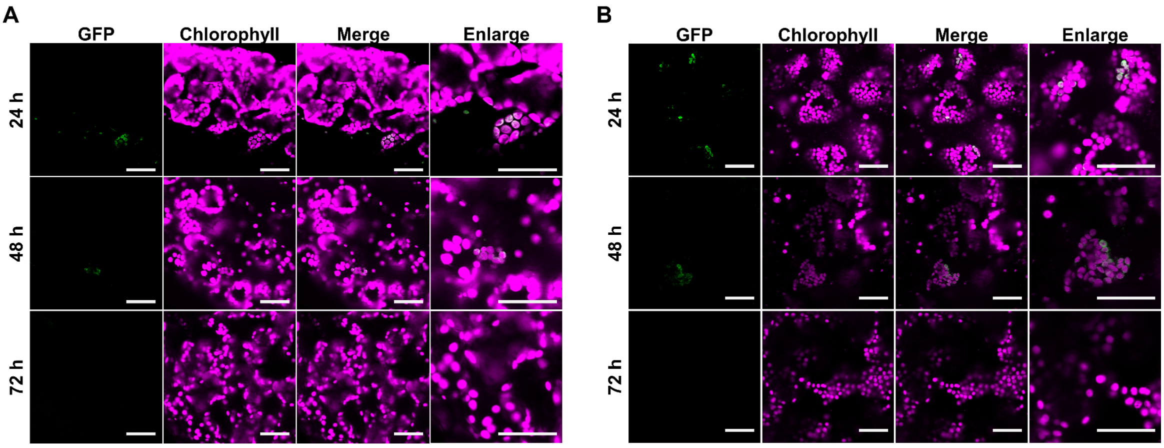

Figure S18. Time course evaluation of CTP/CPP-MC-mediated chloroplast GFP expression in *A. thaliana* cotyledons. CLSM images obtained from (A) ZIL-untreated and (B) -pretreated samples. Scale bars, 40 µm.

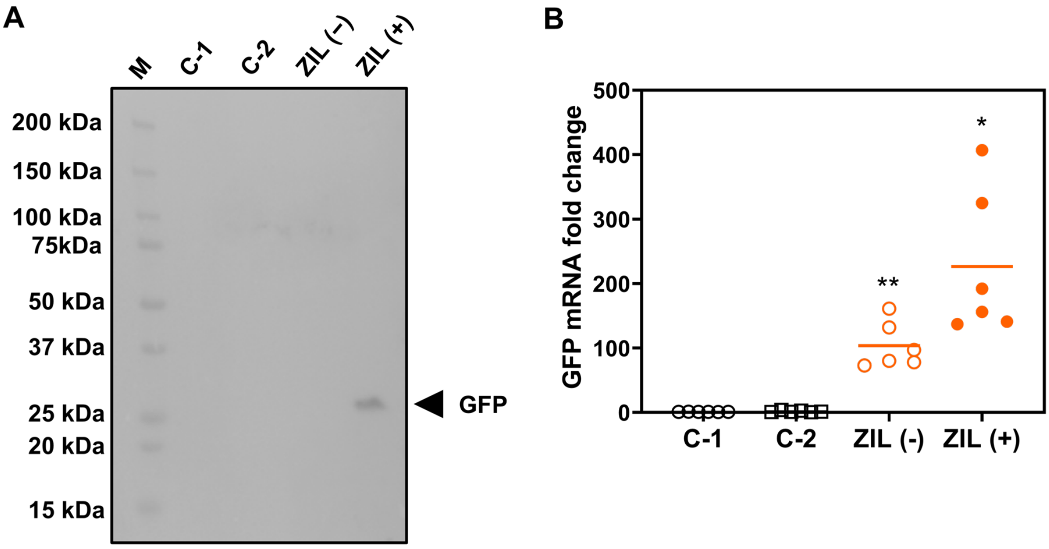

Figure S19. Validation of chloroplast-specific GFP expression in the transfected *A. thaliana* seedlings. (A) Western blot and (B) RT-qPCR analyses of GFP expression in transfected samples under various conditions. C-1, control sample transfected with naked pDNA for chloroplast GFP expression (Prrn-GFP-TpsbA). C-2, control sample transfected with CTP/CPP-MC containing pDNA for nucleus expression (P35S-GFP-Tnos). ZIL (−), samples untreated with ZIL followed by transfection with CTP/CPP-MC containing pDNA for chloroplast GFP expression (Prrn-GFP-TpsbA). ZIL (+), samples pretreated with ZIL followed by transfection with CTP/CPP-MC containing Prrn-GFP-TpsbA. M in (A), molecular weight marker. Statistical significance in (B) was determined by Dunnett's T3 test (n = 6 biological replicates).

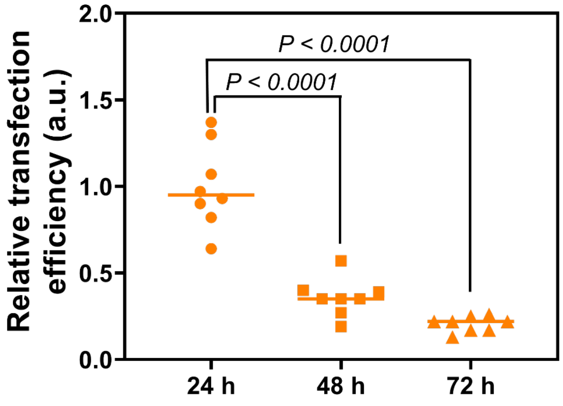

Figure S20. Time course evaluation of Rluc-based transfection efficiency of CTP/CPP-MC in *A. thaliana* cotyledons. Statistical significance was determined by Dunnett's T3 test (n = 10 biological replicates).

Supplementary references

[1] T. Miyamoto, K. Tsuchiya, K. Numata, *Nanoscale* **2021**, *13*, 5679-5692.

[2] C. Thagun, J.-A. Chuah, K. Numata, *Adv. Sci.* **2019**, *6*, 1902064.

[3] K. Kuroda, H. Satria, K. Miyamura, Y. Tsuge, K. Ninomiya, K. Takahashi, *J. Am. Chem. Soc.* **2017**, *139*, 16052-16055.

[4] K. Tsuchiya, N. Yilmaz, T. Miyamoto, H. Masunaga, K. Numata, *Biomacromolecules* **2020**, *21*, 1785–1794.

[5] S. Kalve, K. Saini, K. Vissenberg, T. Beeckman, G. T. S. Beemster, *Bio-protocol* **2015**, *5*, e1592.

[6] X. Liu, J. Li, H. Zhao, B. Liu, T. Günther-Pomorski, S. Chen, J. Liesche, *J. Cell Biol.* **2019**, *218*, 1408-1421.

[7] T. Miyamoto, K. Toyooka, J.-A. Chuah, M. Odahara, M. Higchi-Takeuchi, Y. Goto, Y. Motoda, T. Kigawa, Y. Kodama, K. Numata, *JACS Au* **2022**, *2*, 223-233.

[8] T. Miyamoto, K. Tsuchiya, K. Numata, *Biomacromolecules* **2019**, *20*, 653–661.
